# A Formal Account of Structuring Motor Actions With Sensory Prediction for a Naive Agent

**DOI:** 10.3389/frobt.2020.561660

**Published:** 2020-12-01

**Authors:** Jean-Merwan Godon, Sylvain Argentieri, Bruno Gas

**Affiliations:** Sorbonne Université, CNRS, Institut des Systèmes Intelligents et de Robotique, ISIR, Paris, France

**Keywords:** sensory prediction, sensorimotor contingencies, interactive perception, developmental robotics, bootstrapping

## Abstract

For naive robots to become truly autonomous, they need a means of developing their perceptive capabilities instead of relying on hand crafted models. The sensorimotor contingency theory asserts that such a way resides in learning invariants of the sensorimotor flow. We propose a formal framework inspired by this theory for the description of sensorimotor experiences of a naive agent, extending previous related works. We then use said formalism to conduct a theoretical study where we isolate sufficient conditions for the determination of a sensory prediction function. Furthermore, we also show that algebraic structure found in this prediction can be taken as a proxy for structure on the motor displacements, allowing for the discovery of the combinatorial structure of said displacements. Both these claims are further illustrated in simulations where a toy naive agent determines the sensory predictions of its spatial displacements from its uninterpreted sensory flow, which it then uses to infer the combinatorics of said displacements.

## 1. Introduction

*Autonomous* robots need possess the cognitive capabilities to face realistic and uncertain environments. Classical approaches deal with this problem by giving them *a priori* models of their interaction with their environment. These rely on carefully crafted models of the agent's body (Mutambara and Litt, 1998), its sensors, the environments it will encounter and the nature of the tasks it is setting to perform (Marconi et al., 2011). But said models are notoriously difficult to obtain (Lee et al., 2017), by definition incomplete (Nguyen et al., 2017) and often fail to generalize to interactions varying in unknown spatial and temporal scales. As it has been previously studied, models of an agent sensorimotor apparatus (Censi and Murray, 2012) or of a mobile robot interaction with its environment (Jonschkowski and Brock, 2015) can alternatively be learned. In particular, these capabilities crucially depend on the robot correctly learning its *perception* in that it represents the interface layer between the raw readings of its sensors and its higher level cognitive capabilities, e.g., decision making or task solving layers.

While there certainly is an established practice of mostly treating perception as processing the sensory signal, multiple cues argue that perception can only emerge from the joint *sensorimotor* experience (Noë, 2004). The field of interactive perception, reviewed in Bohg et al. (2017), indeed displays several approaches which roughly adhere to this principle. One particular theoretical framework is that of *Sensorimotor Contingencies Theory* (O'Regan and Noë, 2001) (SMCT for short) which asserts that perception is the mastery of invariant structures in the sensorimotor flow an agent discovers during its interaction with the environment. Their theoretical origin as an abstract, generic cognitive construct lends them desirable properties for robotic applications: namely, they could support bootstrapping the learning of perceptual capabilities in a way that does not depend on the implementation of the artificial agent considered as well as on the environments in which said learning is done. In this regard, it differs significantly from the modern developmental approaches supported by Deep Learning (Ruiz-del-Solar et al., 2018) which rely on specific structural details of neural networks, i.e., the numeric forms of inputs, outputs and activation of the neurons.

Early works on SMCT have been shown to lead to discovery of the color spectrum (Philipona and O'Regan, 2006) and of the dimensionality of ambient space (Philipona et al., 2003, 2004; Laflaquière et al., 2010; Laflaquière et al., 2012) in “naive” agents, which can be extended to that of an internal, path independent, notion of space (Terekhov and O'Regan, 2016). We already proposed different contributions in this field, successively dealing with peripersonal space characterization (Laflaquière et al., 2015), self-contact and body representation (Marcel et al., 2017) and the emergence of a topological representation of sensors poses (Marcel et al., 2019). These works, as well as Laflaquière et al. (2018) and Laflaquière and Ortiz (2019), devote a significant effort to providing formalisms suited to make explicit, and, where applicable, formally prove—not only the processing required to capture the contingencies, i.e., invariants in the sensorimotor flow of the agent, but also the mechanisms by which said contingencies should appear. This is an attempt to pinpoint the exact conditions of validity of the proposed processes in order to deliver on the promises of genericity of SMCT.

Many recent contributions drawing from SMCT revolve around sensorimotor prediction in some way: the ability to discover a sensorimotor prediction is empirically shown to arise from both the temporal structure of the sensorimotor experience (Maye and Engel, 2012) and the spatial coherence of a natural visual environment for a sensor based on a retina (Laflaquière, 2017). Moreover, said ability to predict sensory outcomes has been shown to provide in robots a basis for an egocentric representation of ambient space (Laflaquière and Ortiz, 2019), object perception (Maye and Engel, 2011; Le Hir et al., 2018), action selection (Maye and Engel, 2012), motor control (Schröder-Schetelig et al., 2010), and motor sequence compression (Ortiz and Laflaquière, 2018). Along the “Bayesian brain” approach, predictive processing is even argued to form the mechanistic implementation of sensorimotor contingencies (Seth, 2014). This is very much in line with classical findings in cognitive psychology, both those regarding the physiological implementations of sensorimotor prediction via *efference copies* (von Helmholtz et al., 1925; Sperry, 1950; von Holst and Mittelstaedt, 1950) and how it supports, albeit incompletely, a number of perceptual processes (Bridgeman, 1995; Imamizu, 2010; Pynn and DeSouza, 2012; Bhanpuri et al., 2013), as well as those supporting ideomotor theory (Stock and Stock, 2004) according to which actions are equated to their perceptual consequences from a cognitive standpoint.

This article follows much of the same approach begun in Philipona et al. (2003) and subsequently developed in e.g., Marcel et al. (2017); Marcel et al. (2019), Laflaquière et al. (2018), and Laflaquière and Ortiz (2019). In particular, it sets out to mathematically describe from an *exterior*, “objective,” point of view some properties of the interaction between the robot and its environment which should appear in its sensorimotor flow. To this end we propose a revised formalism building upon the previous instances in these contributions. One notable contribution indeed resides in our proposal remedying their requirement of the agent having a fixed base by transposing the location of sensorimotor contingencies in sets of “displacements” instead of that of motor or sensory configurations. In accordance with the previous remark about genericity, a particular attention is given to the construction of said formalism with assumptions and proofs explicitly detailed. Moreover, the bootstrapping aspect is emphasized throughout the work, much in the spirit of Marcel et al. (2017); Marcel et al. (2019), highlighting the distinction between the points of view of the agent and of the observer in the description of the problems and an explicit discussion of the degree of *a priori* knowledge given to the agent, in terms of both data and computations available to it. There lie two contributions of this article: while the formalism is used to formally describe why and how spatial coherence lead to the discovery of sensory prediction very much like alluded to in Laflaquière (2017) and how this sensory prediction encodes spatial structure akin to that of Terekhov and O'Regan (2016) and Laflaquière and Ortiz (2019), it does so with a greater emphasis put on the precise relations between the algebraic structures at play and with much weakened assumptions about *a priori* capabilities of the agent, much closer to those put forward in O'Regan and Noë (2001). We argue that this formalism unifies and extends those found in previous works; that the formal structures its expressive power makes explicit (e.g., group morphisms between action and prediction) give a conceptual explanation of results previously achieved by more complex means in experimental contexts (Ortiz and Laflaquière, 2018; Laflaquière and Ortiz, 2019); and on a somewhat “philosophical” level that it allows for a clearer picture of the applicability and function of SMCT in the process of bootstrapping perception *via* its systematic distinction of points of view.

The paper is organized as follows: to begin with, we introduce in section 2 all the notations and concepts used for describing the sensorimotor experience. On this basis, section 3 defines the two distinct points of view and enunciates generic properties of the sensorimotor experience that motivate the proposed study of internal sensorimotor prediction. In particular, the equivalence between the combinatorial structures of actions and sensory prediction is proved. Then, some simulations are proposed in section 4 to assess the mathematical formalism through a careful evaluation of each step of the proposed framework. We establish that the spatial shifts mediating the sensory experience of a naive agent allow it to determine the sensory outcome of particular actions, in particular those corresponding to displacements of the agent. Further, we show that the ability to predict said outcomes can be used as a proxy to the hidden combinatorial structure of its motor actions. We argue that the theoretical focus adopted in this work provides some new valuable insight into the mechanisms supporting these results, as well as several similar findings presented in aforementioned related works.

## 2. Defining a Formalism for Sensorimotor Interaction

This first section aims at expanding several previous results in *Interactive Perception* as obtained for example in Bohg et al. (2017). These have made use of several classical objects, such as the *pose* (or *working*) *space* and the *forward* (either geometrical or sensory) *maps*, at times rearranging their definitions or making them more precise to allow for formal proofs to be derived. Such work is followed upon in this contribution, with a somewhat significant overhaul of the formal definitions. This section is thus devoted to the definitions of the terms we will use to describe a sensorimotor problem, showing during the exposition how they appear in a simple classical example and how they differ from previous theoretical formulations. We then leverage these definitions to propose and prove new perceptive bootstrapping algorithms in the following section.

### 2.1. Motor Actions

As a first step, this subsection is devoted to the introduction of all the notions and definitions of the *motor* side of the proposed sensorimotor framework. After highlighting the limitations of the previous approaches, as seen in e.g., Marcel et al. (2017) and Laflaquière et al. (2018), we show how to reparameterize the sensorimotor interaction by introducing motor actions. Their definition and properties are then carefully discussed.

#### 2.1.1. A Look Back to Previous Formalisms

Let us consider in all the following an agent endowed with motor and sensing capabilities. In the referenced previous contributions, the sensorimotor interaction is defined by the *internal* motor configuration **m** and the sensory configuration **s** of this agent, which lie, respectively into some sets M and S. Both of them define the *internal* agent configuration (**m**, **s**), i.e., the sensorimotor flow the agent has access to. There is a clear dependency between the sensory and motor configurations that can be captured by the sensorimotor maps ψ:M×E→S, such that ψ(**m**, ***ϵ***) = **s**, where ***ϵ*** ∈ E represents the state of the environment. As said in the introduction, other contributions already exploited this kind of parameterization (Philipona et al., 2003; Laflaquière et al., 2015; Marcel et al., 2017). In all these contributions, only fixed base agents are considered, since a single internal motor configuration m∈M is only mapped to a single sensory configuration s∈S for a fixed environment configuration ***ϵ***.

**Figure 1:**
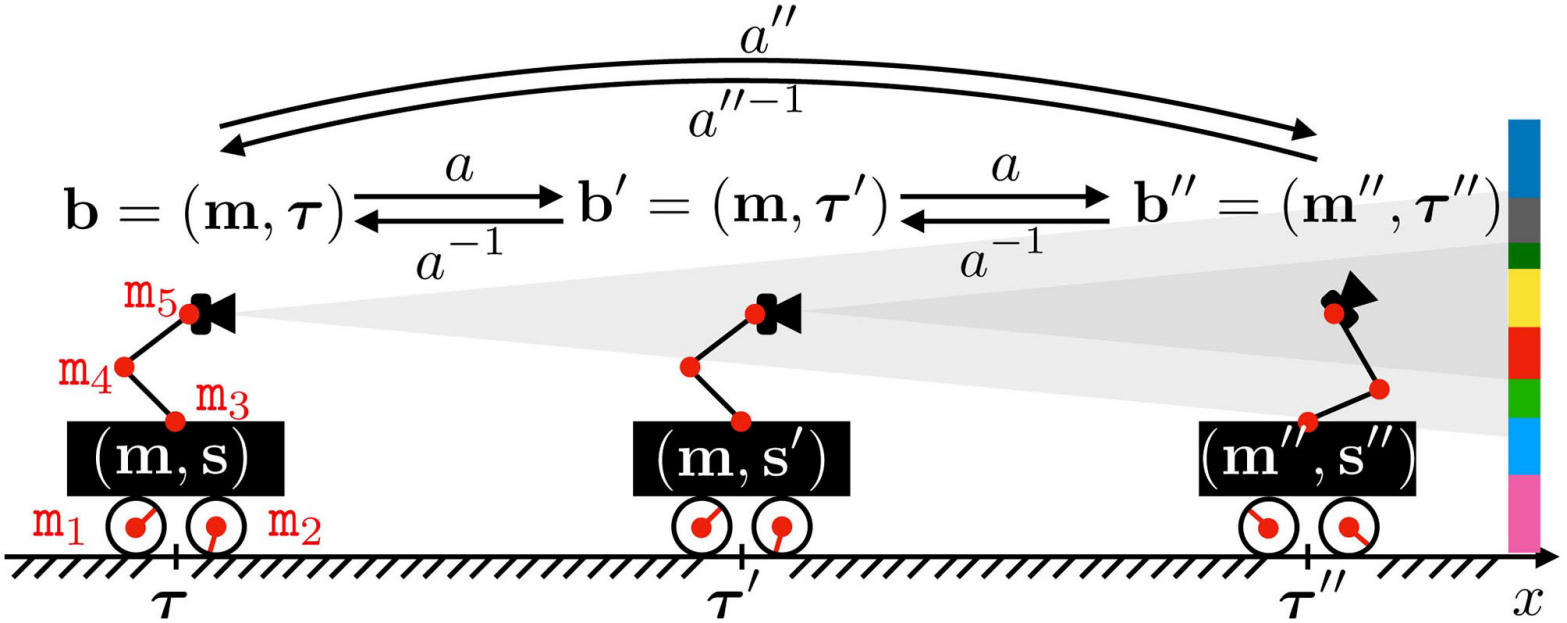
Illustration of the motor actions effects. The agent actuator states m_*i*_ are regrouped into its motor configuration **m**, while **s** denotes its sensor output. Both define the internal agent configuration, i.e., the sensorimotor flow the agent has access to. The agent position and orientation in space is captured by ***τ***, which together with **m** makes the absolute configuration **b**, partially unknown to the agent. Note that one motor configuration **m** can be associated to distinct sensory outputs **s** and **s′** provided a displacement in space from ***τ*** to ***τ***′, thus preventing the existence of a mapping **m**↦**s**. The agent may also perform an action *a* to modify its absolute configuration. One such action *a* is partially represented, mapping **b** to **b′** = (**m′**=**m**, ***τ***′), and **b′** to **b″**. Note that it induces a rigid displacement in the first case, while a change of posture occurs in the second. Also depicted are the inverse action *a*^−1^ of *a* and the combination *a*″ obtained by repeating *a* twice.

To illustrate this point, Figure 1 represents a 2D-agent able to translate itself only along one dimension *x*. This agent is able to move inside an environment made of colored walls thanks to five rotating joints whose states m_*i*_, *i* = 1, …, 5, are captured in its motor configuration **m** = (_m_*i*_)*i*_ (where the second *i* subscript in (_._*i*_)*i*_ denotes the collection being taken with *i* for ranging variable). To begin, let us consider the case where m_1_ and m_2_ are fixed, so that the agent is only able to move its arm supporting a camera-like sensor generating a sensation **s**, i.e., **m** is restricted to (m_3_, m_4_, m_5_) only. In such a scenario, one has a fixed base agent for which each motor configuration **m** can be mapped to one corresponding sensor pose, which is itself mapped to a sensation **s**. This simple statement allows to build structures in M by exploiting only the sensorimotor flow (**m**, **s**), structures that can be leveraged to build an internal representation of the agent body; they can be further refined into a representation of its peripersonal space (Marcel et al., 2017; Marcel et al., 2019). In these works, **m** carries all spatial data, possibly with some redundancy, about the coupling between the agent and its environment: the combination of states **m** and **e** is sufficient to determine the resulting sensory output **s** (as described by the formal sensorimotor map ψ).

However, what would happen if the same agent was able to perform translations in its environment? Let us now focus on the case where *all* motor states m_*i*_ are actually used, as depicted in Figure 1. Indeed, one can imagine a case where the agent moves in its environment along the *x* axis from (external) position ***τ*** (with internal configuration **m**) to ***τ***′ (same **m**). In this case, the sensor samples two different parts of the color wall so that its generated sensations **s** and **s**′ from these two different positions are different. Then two identical internal configurations **m** give two different sensations: there is no more mapping between **m** and **s**, and all the mathematical developments performed in previous works can no longer apply. Therefore, it seems necessary to generalize these formalisms to cope with agents able to move freely in their environment. In this paper, one proposes a *variational* formulation of motor actions to deal with this issue. Importantly, the term *variational* refers in all the following to the focus given on specific sequences of states (e.g., motor, sensory, or external states) rather than any specific one of said states. It is introduced in the next subsections.

#### 2.1.2. Dealing With Mobile Agents: Reparameterizing the Sensorimotor Interaction

From previous arguments, the internal motor configuration **m** can not be mapped unambiguously to sensations without additional considerations. If one still insists on having a functional relation between motor data and sensations, one then needs to enrich the initial motor set. In this paper, one proposes to introduce some superset B of M as initial parameter space. This new set B can be thought of as the set of all *absolute* configurations **b** made of pairs (**m**, ***τ***) where **m** is the internal motor configuration and ***τ*** represents an absolute measure of the pose of the agent in its ambient space (which would most commonly be position and orientation in 3D space). While posture or proprioception give **m** a definite meaning, one should instead only think of ***τ*** as a choice of reference frame in space. Indeed for spaces much like ours it ought to be somewhat arbitrary since any “displacement” from pose ***τ*** to ***τ***′ could be instead realized as an opposite motion of the whole ambient space like for compensatory movements as initially introduced in Poincaré (1895) and dealt with in Philipona et al. (2003). This equivalency argument has been mentioned in previous contributions as a possible way to deal with mobile agents as proposed in this contribution. While this could be formalized in a consistent way, either from a quasi static or variational perspective, we argue the proposed point of view offers usability advantages, like for multi-agent situations (where space should emerge as a shared common playground), an easier formulation of compensability, or a clear separation between the internal and external points of view. It is important to understand that the agent itself has no knowledge of the current absolute configuration **b** of its interaction with its environment, retaining the same hypotheses about a priori structure. However, we may then consider the sensorimotor map as a function ψ:B×E→S instead of ψ:M×E→S to account for possible displacements in the environment. Defining such a new set B allows then to introduce the notion of *external* agent configuration as the tuple (**b**, **s**). As such, two different points of view must be stressed out: (i) the external point of view (i.e., coming from the designer of the system) will allow to characterize some properties of the agent interaction with its environment (through modelization, hypotheses, etc.), and (ii) the internal point of view which represents which data and concepts are available to the agent for its operations. This specific point is discussed in section 3.1.

Coming back to Figure 1, the agent moves to three successive absolute configurations **b**, **b**′, and **b**″. All of them are now different, which was not the case of the internal motor configurations: introducing **b** ∈ B apparently solves the issue raised at the end of section 2.1.1. Let us now explain how the agent actually reaches some given absolute configuration **b**.

#### 2.1.3. Going Variational: Introducing Motor Actions

As explained previously, the agent has no direct access to the configuration data **b**: it cannot know where it *is* in B. Instead we suppose it starts with some (very limited) knowledge of how it *moves* in this set, i.e., it is capable of performing some moves in B and of comparing any two moves for equality. To this end, we propose to introduce some new set A behaving in the following manner: an element a∈A can be applied to any absolute configuration **b** ∈ B to give a new configuration **b**′ = *a***b** = *a*(**b**). Therefore, *a* can be seen as a function B→B. We will usually denote $\text{b}\overset{a}{\to }{\text{b}}^{\prime }$ this situation, and call *a* a *motor action*. Such a definition for “actions” differs from many intuitions since it is restricted to quasistatic differences in posture and position; it does not account for a notion of dynamical effort exerted by actuators. In particular, no dynamical effects are considered at this level and no specification is made of the precise motor path taken from **b** to *a***b**. Instead, only these pairs of related (**b**, *a***b**) endpoints are relevant to characterizing any action *a*. This constitutes a present limitation pervading much of similar works to which a future—and assuredly significant—contribution shall be devoted.

Now as we intend to represent the way in which the agent can move in its environment, one can take for granted the existence of a special action e∈A that verifies $\forall \text{b}\in B,\;\text{b}\overset{e}{\to }\text{b}$: the agent may decide to stay still. Note that for certain systems, e.g., drones or bipedal walkers, this is distinctly different from doing nothing since constant posture and position must still be maintained. Moreover, considering it is able to do any moves *a* and *a*′, it may then chain them in one single move $a^{\prime \prime }=a^{\prime }a\in A$ which satisfies 
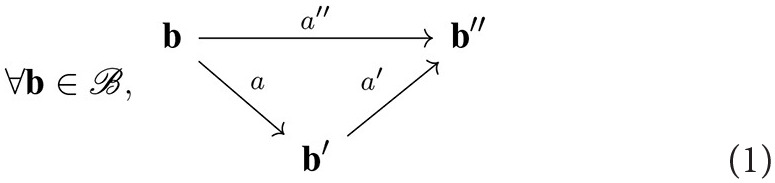


so that A naturally carries the structure of a *monoid*. Remark that this composition operation is necessarily associative since motor actions are assumed to behave as functions B→B. In the following, we will further restrict ourselves to the case where individual actions are *reversible*, that is for any action *a* there exists an action *a*^−1^ such that 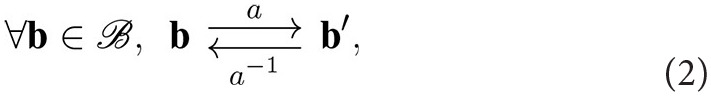


making A into a *group*. Seeing as how actions can be thought of as mappings B→B, a necessary (and sufficient) condition is for all mappings in A to be bijective. It is clear that this assumption of invertibility may not apply in some experimental contexts, e.g., an agent may jump down a height which it cannot jump up. This constitutes a current limitation of the proposed framework, although several factors may limit its severity. In this example, even if the agent cannot jump up the height directly, it may still find a sequence of actions allowing it to climb back up to its original position. Corresponding to the definition of Equation (2), this would result in an inverse action in the formal sense, as illustrated later in section 4.3.3.

Figure 1 illustrates these notions, with the agent moving from external configuration **b** to **b**′ through an action *a*. This action, applied at **b** = (**m**, ***τ***), happens to produce a translation of the agent so that its internal motor configuration finishes at the same **m**. Note that the agent would be able to return back to its initial absolute configuration by applying the inverse action *a*^−1^ of *a*. Moreover, since *a* is a function defined on the whole B set, the same action can be applied at **b**′ = (**m**, ***τ***′) to reach a third configuration **b**″ = (**m**″, ***τ***″). This time, the same action *a* has conducted to a global displacement of the agent in the environment, combined with a change in its internal motor configuration. Indeed, while it represents cases which are mostly avoided for practical reasons, it is not required for *a* to only depend on **m** in the general case: the outcome of the same action *a* may depend on the position ***τ*** of the agent in the environment. Finally, the agent would have been able to move from **b** to **b**″ by applying the action *a*″ = *a*^2^, as per Equation (1).

With these structure assumptions, for a given subset of *motor primitives* $A^{\prime }\subset A$ available to the agent, we can search for the set of composed moves the agent can actually reach by iteration of its known ones. We shall say an action a∈A *decomposes over* $A^{\prime }={\left\{a_{i}\right\}}_{i\in I}$ if it can be written in the form

(3) $$\begin{array}{l} a=a_{i_{n}}\ldots a_{i_{1}}=\underset{1\leq k\leq n}{\prod }a_{i_{k}},i_{k}\in I \end{array}$$

This represents a formal property functionally similar to that of compositionality of motor trajectories, with $A^{\prime }$ filling for actions the role of primitives (Flash and Hochner, 2006). Indeed, the interest of these decompositions appears because the effect of composed moves on motor configurations boils down to the effects of its components as per the following diagram: 
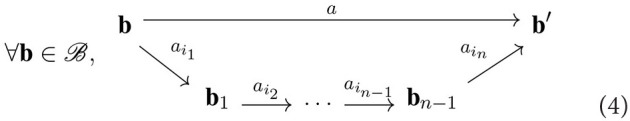


In the example in Figure 1, it may well be that the agent can move to any configuration **b**, i.e., that its action set is $A={\mathbb{R}}^{5}$ (for the five possible angular increments of its five joints). But it may also be restricted to a limited set of moves, for example if it only can send discrete commands to its joints. For instance, if each actuator is a stepper motor, then its action set turns into $A={\mathbb{Z}}^{5}$. In this case, *a* would be written as the tuple (Δ_*q*_*i*_)*i*_, *i* = 1, …, 5, where Δ*q*_*i*_ ∈ ℤ is the *i*th motor increment expressed in step increments. Consequently, any action *a* would decompose over $A^{\prime }={\left\{a_{i}\right\}}_{i}$ where action *a*_*i*_ corresponds to adding one step to the *i*th actuator. In this specific case, while A is infinite, it is sufficient for the agent to know the five motor primitives *a*_*i*_ to generate any action a∈A. This is very similar to the notion of reducing a (finite dimensional) vector space, which is usually infinite, to the very finite subset of a generating set or if possible a base. However it can be proven that any finite subset of ℝ will not generate it as a group, and that it will often only generate a discrete *kℤ* subgroup. This occurs in the proposed simulations in section 4, where all combinations of a finite subset of starting actions lead to the discovery of a discrete generated action group.

### 2.2. Grounding Sensations in Space

The previous subsection was devoted to the introduction of actions on the motor side of the proposed sensorimotor framework. This subsection accordingly deals with the *sensory* side of it, and more particularly with its relation to a persistent “space” which was entirely absent from previous considerations. After a more precise definition of the meaning of “environment configuration,” the link between local perception and spatial considerations is formalized. This will constitute the root of the theoretical developments proposed in the next section.

#### 2.2.1. Decoupling Space and Environment: The *Where* and the *What*

In previous works, a traditional way for parameterizing the environment was to introduce the environment configuration ***ϵ***. The meaning of such a variable was often left unspecified, almost without any formal semantics linking it to the sensorimotor experience of the agent (Laflaquière et al., 2015). In this paper it is proposed to stress the difference between the ambient geometrical *space*—in which sensorimotor experience occurs—and the *environment* itself—that is the state of “things” lying in this space. The former takes the form of some set X endowed with a spatial structure as encoded by a group G(X) of *admissible transformations*. These spatial transformations are mappings X→X preserving some “geometry” of X. The most common illustration is the usual affine geometry of ℝ^3^ given by the group ${\text{SE}}_{3}\left(\mathbb{R}\right)={\text{SO}}_{3}\left(\mathbb{R}\right)⋊{\mathbb{R}}^{3}$ of its rigid transformations, made of 3D rotations, translations and their compositions. On this basis, one chooses to particularize a “state of the environment” as a valuation that maps each point of X to its corresponding physical properties, such as temperature, color, luminance, etc. These states are therefore best represented as functions ϵ:X→P where P is a set describing the different physical properties the agent can observe. Consequently, ϵ(**x**) represents the observable physical properties at point x∈X. We will henceforth denote E the set of *environment states*, i.e., a set of such functions ϵ.

**Figure 2:**
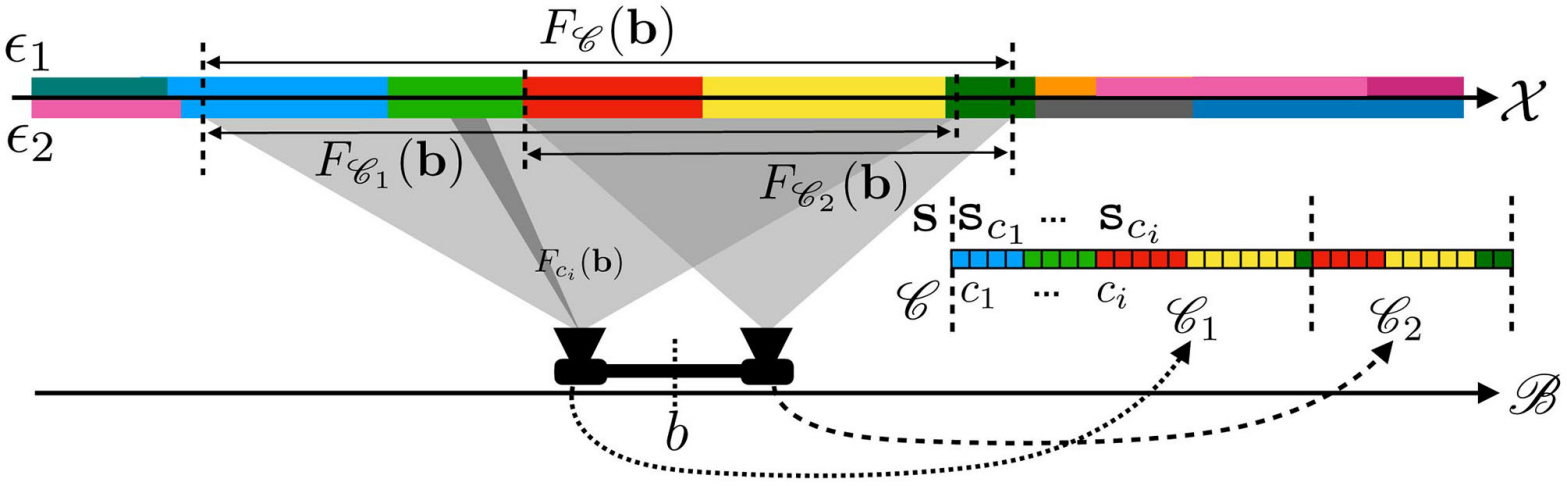
Illustration of the receptive fields for a sensor made of two rigidly linked cameras at configuration **b**. Each pixel *c*_*i*_ of either camera produces a sensory value s_*c*_*i*__ in the overall sensory array **s** explained only by a small subset of space *F*_*c*_*i*__(**b**). The same applies for both cameras, thus explaining how a sensation for the agent can be explained by the perception of a subset of space.

Figure 2 illustrates these considerations. In this simple case, the geometrical space X is monodimensional, represented as an axis where each point **x** is assigned a color through a function ϵ_1_ or ϵ_2_. Interestingly, one can now distinguish points in X on which the physics described by different environment states ϵ_1_ and ϵ_2_ locally coincide, as far as the agent is able to observe this coincidence. Particularizing the former unspecified ***ϵ*** state to a function ϵ of the spatial variable will allow to express new properties of the sensorimotor experience, as in the next subsection.

#### 2.2.2. Local Perception and Receptive Fields

Now that we have formally defined what is “out there” from an external point of view, let us now focus on the sensory capabilities of the agent. On this specific point, most previous contributions were considering the full sensory output as atomic data: although it is implemented as a possibly high dimensional vector, elements and subarrays were generally kept from scrutiny. On the contrary, we now take interest at the subarray level and accordingly adapt the formalism. Therefore, in all the following the sensorimotor map is written as $\psi_{c}:B\times E\to S_{c}$ where the *c* subscript outlines that the sensory map is explicitly written for a sensory element *c* (or *sensel*, i.e., one pixel for a camera, the cochlea cell coding for one sound frequency, etc.). Thus, the sensorimotor map ψ_C_ for the entire sensory apparatus is made of the aggregate of all sensels along $\psi_{C}:B\times E\to S=\prod_{c\in C}S_{c}$ with C the set of all sensels.^1^ An illustration of these points is proposed in Figure 2 for a similar agent endowed this time with two cameras so as to better show the descriptive capabilities of the formalism. In this case, the sensels *c*_*i*_—each depicted as elements in a color array—represent the pixels of either camera. Separate sensors in the apparatus thus appear as sub-arrays in C: the first (resp. second) camera is figured by C_1_ (resp. C_2_). Note that this decomposition of C as C_1_ ∪ C_2_ directly comes from our external understanding of the agent structure (i.e., with one camera corresponding to one set of sensels, i.e., one sensor). One could have selected others sub-arrays to form a distinct set of (virtual) sensors not necessarily corresponding to their (physical) implementation on the agent.

With space and sensors made formally precise we can now proceed with the (spatial) *receptive field* of sensor C′ ⊂ C, that is a region of space which environment state suffices to determine the output of C′. This region as a subset of X should naturally depend on the current configuration **b** since moving causes one's sensors to sample new parts of space, so that it takes the form of a map $\text{b}\in B\mapsto F_{C^{\prime }}\left(\text{b}\right)\subset X$. Then, its characteristic property is

(5) $$\begin{array}{l} \forall \epsilon_{1},\epsilon_{2}\in E,\forall \text{b}\in B,\\ \epsilon_{1|F_{C^{\prime }}\left(\text{b}\right)}=\epsilon_{2|F_{C^{\prime }}\left(\text{b}\right)}\Rightarrow \psi_{C^{\prime }}\left(\text{b},\epsilon_{1}\right)=\psi_{C^{\prime }}\left(\text{b},\epsilon_{2}\right). \end{array}$$

Figure 2 represents some of the receptive fields for the two cameras agent. The first one, *F*_*c*_*i*__(**b**), is the receptive field of a single sensel/pixel *c*_*i*_ ∈ C. The receptive fields *F*_C_1__(**b**) and *F*_C_2__(**b**) of each camera can be obtained as the union of the receptive field *F*_*c*_*j*__(**b**) of their respective pixels. In the same vein, the overall receptive field of the agent *F*_C_(**b**) is also given by *F*_C_1__(**b**) ∪ *F*_C_2__(**b**). From the same figure, it is clear that even if ϵ_1_≠ϵ_2_ (since there are areas of different colors on the X axis), the sensation captured by the agent is the same since the aforementioned differences are restricted to areas of space unseen to the agent.

It is important to notice that this is the formal step where the notion of receptive field formalizes an implicit relation between the sensations of the agent and spatial structure. This constitutes one fundamental property sufficient to leverage spatial knowledge from the agent interaction with its environment. The application of these theoretical elements is proposed in the next section.

## 3. A Zero-th Layer of Sensorimotor Contingencies: Spatial Regularities Through Variations

In this section, we proceed by describing how the formal elements from section 2 can be arranged to enunciate some interesting properties of the sensorimotor interaction. First, to keep in line with considerations of minimalist bootstrapping, the assumptions we use relative to the model of knowledge of the agent are discussed, and compared to that of previous contributions. Then, the definitions provided in the previous sections are used to isolate conditions where the spatial structure of the receptive fields can be leveraged, in particular *via* a certain class of “conservative” actions which are themselves defined. We prove that under these conditions a naive agent may achieve the determination of sensory prediction functions for said conservative actions, and that the algebraic structure of these prediction functions matches that of their actions. The corresponding results are of two distinct but equally important sorts: some, taking the viewpoint of an external observer, assert that certain particular objects of interest (such as a *sensory prediction function*) *exist*; others guarantee these objects to be *computable* in the boundaries set by our model of knowledge. This endeavor is made in an effort to keep *a priori* knowledge to a minimum, and these proofs are generally of a constructive nature.

### 3.1. Model of Knowledge of the Agent

In the authors' previous works (Marcel et al., 2017; Marcel et al., 2019), sensorimotor interaction occurred as a sequence of (generally discrete) steps where at each point, the agent could access both its proprioception m∈M (seen as an array of current joint configuration states) and its corresponding exteroceptive array **s** = ψ(**m**, ***ϵ***). These sensory arrays were then compared for equality (and for equality *only*) *as total vectors*, that is the agent may not access the vectors component by component. It is crucial to note that, much like in the referenced articles—and following the argument that “there is no *a priori* reason why similar neural processes should generate similar percepts” as found in O'Regan and Noë (2001)—we will assume here that the sensory signals are *uninterpreted* in the very strong sense that they retain no other structure than equality. This presents an *a priori* significant hurdle since this includes, e.g., order comparisons, substractions, metric structures and precludes us from using objects, such as gradients or clusterings, which are required in almost all comparable works (Censi and Murray, 2012; Montone et al., 2015; Laflaquière, 2017 among others). This knowledge was then used for example to compute set-theoretic motor kernels (Marcel et al., 2017) which were shown to be a structural invariant of the sensorimotor interaction (Marcel et al., 2019). By contrast, in this paper slight modifications are applied. Indeed, from the external point of view we now have B as a functional analog to the previous M, that is the set of “parameter” data that entirely determines the state of the interaction between agent and environment. However, as the definition of B refers to some explicitly *external* data [i.e., the ***τ*** in **b** = (**m**, ***τ***)], we cannot assume its knowledge from the point of view of the agent. We could however elect, on the same basis previous contributions used, to assume internal knowledge of the **m** part of **b** = (**m**, ***τ***). Instead, we even assume no direct access to “proprioceptive” data and treat it as unknown to the agent. Our hypothesis is that the agent should learn to isolate what part of its proprioception lies in its unified sensory array **s** from the statistics of its sensorimotor experience.

As for remedies, it is instead where a variational approach, as defined in section 2.1.1, is preferred: while configuration data represented by **b** ∈ B still exists as an *external* object, the agent may only choose a *motor action* a∈A which, applied at **b**, yields the following configuration **b**′ = *a***b** = *a*(**b**). The agent is therefore given the capacity to compare any two elements of A for equality, so that it may tell whether at any two steps of its sensorimotor experience it performed two identical or distinct actions. Moreover, much deeper change in knowledge occurs at the level of sensory readings: in the following we not only ask that the agent be able to compare its entire sensory output **s** = (_s_*c*_)*c* ∈ C_ for equality as a vector, but that it also can check for equality two values of any given sensel. That is, for every sensel *c* ∈ C, for every values s_*c*_, ${\text{s}}_{c}^{\prime }$ this sensel may output, the agent may test whether ${\text{s}}_{c}=={\text{s}}_{c}^{\prime }$. In this contribution, it will be further assumed that the values output by distinct sensels are themselves *a priori* comparable for equality. While it is a common property in many classical applications, this limitation has been partially tackled in Laflaquière (2017) via sensory prediction. However, this solution relies on clustering methods implicitly exploiting structure assumptions we do not yet consider available. Therefore, this remains a current limitation of our approach which will be addressed in a future ongoing work.

### 3.2. Sensorimotor Binding: A Marketplace for Spatial Information

The formalism introduced in section 2 makes space appear as a variable in the sensorimotor equations via the receptive field, which we will use in this section to prove that under some reasonable assumptions we can talk about the spatial information content of a sensory signal. This in turn is used to form the basis of a *sensory prediction* the agent can use to try and infer the sensory consequences of its motor actions, mirroring the psychological construct of forward sensory model which is at the heart of ideomotor theories. This is the core idea we will further develop in the simulations of section 4 to see how a naive agent can derive such a prediction function from its sensorimotor flow.

Recall that for any given sensel *c* ∈ C and environment state ϵ ∈ E, we introduced *F*_*c*_ the receptive field of sensel *c* as the function which given agent configuration **b** ∈ B yields X'$=F_{c}\left(\text{b}\right)\subset X$ the minimal region of space which entirely determines the output of ψ_*c*_. Therefore, we can write

(6) $$\begin{array}{l} \forall \text{b}\in B,\forall \epsilon \in E,\psi_{c}\left(\text{b},\epsilon \right)=f_{c}\left(\epsilon_{|F_{c}\left(\text{b}\right)}\right), \end{array}$$

where *f*_*c*_ is a “sensitivity” function (or filter) which converts the physical properties of environment sampled into a sensory output, both selecting to which property the sensor reacts and how. Equation (6) describes the sensorimotor dynamics by dissociating the spatial dependency (which is given by *F*_*c*_) and the sensitivity one (as seen with *f*_*c*_), so that the observer can now speak of sensels that *look at the same region of space*. Let us then consider a particular condition, in which for a given action *a*, some sensel *c*_*i*_ samples *after* *a* the same point some other sensel *c*_*j*_ was sampling before the agent began to move. This is formally described by the relation

(7) $$\begin{array}{l} \forall \text{b}\in B,\;F_{c_{i}}\left(a\text{b}\right)=F_{c_{j}}\left(\text{b}\right). \end{array}$$

This situation where the spatial difference between two sensels can be bridged by a displacement of the agent over time to make their respective sensory experiences coincide has already proven to yield interesting structures as in Montone et al. (2015) and Laflaquière (2017). However, these works largely dealt with the geometry of sensels and sensors, while we aim to elaborate on how this relates to the structure of actions. As for us, to have this relation apparent to the agent we also require that the output of these particular sensels be comparable, as already discussed in section 3.1. In the strictest sense, this can be by requiring that their sensitivity functions *f*_*c*_*i*__ and *f*_*c*_*j*__ are equal. It follows that

(8) $$\begin{array}{l} \forall \text{b}\in B,\;\forall \epsilon \in E,\\ {\;\psi }_{c_{i}}\left(a\text{b},\epsilon \right)=f_{c_{i}}\left(\epsilon_{|F_{c_{i}}\left(a\text{b}\right)}\right)\\ \;=f_{c_{j}}\left(\epsilon_{|F_{c_{j}}\left(\text{b}\right)}\right)=\psi_{c_{j}}\left(\text{b},\epsilon \right). \end{array}$$

While there are reasons to hope that a working relation could be found even for dissimilar *f*_*c*_*i*__ and *f*_*c*_*j*__, one should remember that at the moment the outputs of sensels *c*_*i*_ and *c*_*j*_ lie in some sets totally devoid of structure. Therefore, even though a conversion function *C*_*i, j*_ such that ψ_*c*_*i*__(*a***b**, ϵ) = *C*_*i, j*_(ψ_*c*_*j*__(**b**, ϵ)) might exist, we would lack the means to represent it in any way but the collection of the related sensory outputs, e.g., as opposed to the already resource heavy clustering done in Laflaquière (2017).

**Figure 3:**
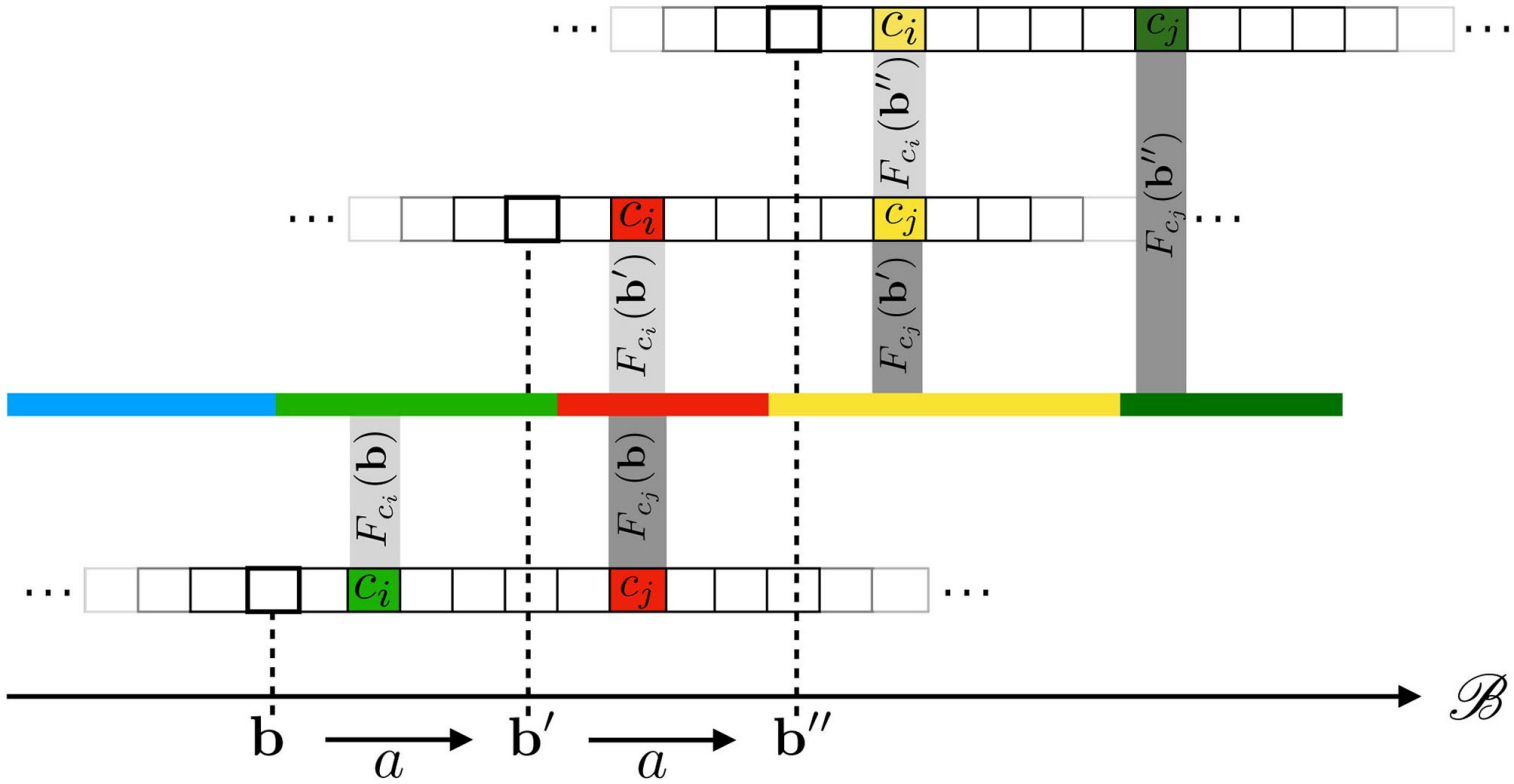
Illustration of how the underlying 1D space induces transitions between cross-sensel outputs. In this case, under action *a*, sensel *c*_*i*_ takes the place of sensel *c*_*j*_: the output of *c*_*i*_ after *a* (red) is the same as the output of *c*_*j*_ before *a* (red). The same applies when performing *a* a second time: the yellow color is transferred from *c*_*j*_ to *c*_*i*_.

Equations (7) and (8) are both illustrated in Figure 3, where a 1D (infinite) pixel array is placed in front of a 1D colored line along which the sensor can translate itself thanks to actions *a*. Equation (7) is captured by the fact that both receptive fields *F*_*c*_*j*__(**b**) and $F_{c_{i}}\left({\text{b}}^{\prime }\right)$, drawn as two rectangular shapes, project on the same area on the environment. Then, Equation (8) explains how, provided the environment state ϵ at these locations stays constant through any one execution of *a*, it causes both sensels to actually generate the same sensory (red) output. It is clear that the spatial relation being forwarded to sensory transitions depends on the sensels actually outputting the same (red) value. This might be argued to be a restrictive assumption. Nevertheless, being able to deal with different sensitivity functions is a sizable development to which an ongoing contribution shall be devoted. To conclude, a key point here is that a property entirely defined from the external point of view through receptive fields is accessible from the internal one by the constraints it imposes on the sensels outputs values during exploration. Equation (8) therefore shows how space, insofar as it is common to all sensels and actions, makes this phenomenon of shifts of receptive fields into an observable contingency of the agent's sensorimotor experience.

### 3.3. A Motor and Sensory Account of Spatial Conservation

#### 3.3.1. Conservation Through Permutation: Conservative Actions

The result obtained in the previous subsection exhibits an important property making internally available spatial matching between receptive fields at different timesteps of motor exploration. But given that the actual motor exploration follows the algebraic structure of actions A, it still remains to be shown how these two structures are consistent. This can be made apparent by introducing *conservative actions* as those *a* of A for which *all* sensels of the agent exchange the places they sample: there is conservation of the (spatial) information available. In terms of the formalism, a∈A is conservative if it verifies

(9) $$\begin{array}{l} \forall c\in C,\exists c^{\prime }\in C\text{ such that }\forall \text{b}\in B,F_{c}\left(a\text{b}\right)=F_{c^{\prime }}\left(\text{b}\right), \end{array}$$

generalizing somewhat Equation (7). This characterization makes apparent that many actions can't be conservative: for example, “turning back” may only be conservative for the rare agent that “sees” precisely as much forwards as it does backwards. In fact, the spatiality of the condition on receptive fields makes it so that all readily found conservative actions correspond to displacements of the body of the agent. In the following, “$\forall \text{b}\in B,F_{c}\left(a\text{b}\right)=F_{c^{\prime }}\left(\text{b}\right)$” will be shortened to the more legible $c\overset{a}{\to }c^{\prime }$, and *c* (resp. *c*′) is said to be the *predecessor* (resp. *successor*) of *c*′ (resp. *c*) by *a*. It is proven in Appendix 1 that for conservative actions *a*, the relation $\overset{a}{\to }$ can be made into a successor function

(10) $$\begin{array}{l} \sigma_{a}:C\to C\\ \;c\mapsto c^{\prime } \end{array}$$

where $c^{\prime }=\sigma_{a}\left(c\right)$ is a sensel verifying $c\overset{a}{\to }c^{\prime }$. Therefore, conservative actions can equivalently be thought of as *permutation of sensels*. Importantly, conservative actions provide a natural framework for exploiting Equations (7) and (8) during motor exploration. Indeed, it is proven in Appendix 2 (see the Supplementary Materials) that conservative actions form a *subgroup* $A_{C}\subset A$ for its composition operation. That is to say, chaining conservative actions yield other actions which are necessarily conservative, and the inverses of conservative actions are themselves conservative.

At this stage, it has been shown how the spatial property of permutation of the receptive fields relates to the intrinsic motor structure of the agent. However, this does not suffice to make this group structure of conservative actions accessible to the agent given the *a priori* knowledge we discussed in subsection 3.1, since the dependency of the sensorimotor process on the spatial variable is implicit. We must therefore go through one final step to relate the available informational content (i.e., sensory reading) to the motor structure.

#### 3.3.2. From Permutation to Prediction: Making It Into Sensory Territory!

Let us consider the agent at any point (**b**, **s**) of its sensorimotor experience. Its sensory output is **s** = ψ_C_(**b**, ϵ) = (_s_*c*_)*c* ∈ C_, and for any action *a* it may perform this sensory output should shift to **s**′ = ψ(**b**′ = *a***b**, ϵ) provided the environment state stays constant throughout the action. If we now restrict ourselves to the case of conservative actions, we get

(11) $$\begin{array}{l} {\text{s}}^{\prime }={\left({\text{s'}}_{c}\right)}_{c\in C}\\ \;={\left(\psi_{c}\left(a\text{b},\epsilon \right)\right)}_{c\in C}={\left(f_{c}\left(\epsilon_{|F_{c}\left(a\text{b}\right)}\right)\right)}_{c\in C}\\ \;={\left(f_{c}\left(\epsilon_{|F_{\sigma_{a}\left(c\right)}\left(\text{b}\right)}\right)\right)}_{c\in C}={\left(\psi_{\sigma_{a}\left(c\right)}\left(\text{b},\epsilon \right)\right)}_{c\in C}\\ \;={\left({\text{s}}_{\sigma_{a}\left(c\right)}\right)}_{c\in C} \end{array}$$

so that performing motor action *a* only results in a permutation of the components of the sensory output. This permutation is exactly σ_*a*_, and therefore is a constant of the agent which does not depend on the actual current configuration (**b**, ϵ). Equation (11) shows that any *conservative* action $a\in A_{C}$ corresponds to a *sensory* function

(12) $$\begin{array}{l} {\;\Pi }_{a}:S\to S\\ {\left({\text{s}}_{c}\right)}_{c\in C}\mapsto {\left({\text{s}}_{\sigma_{a}\left(c\right)}\right)}_{c\in C} \end{array}$$

which verifies the property

(13) $$\begin{array}{l} \forall \text{b}\in B,\forall \epsilon \in E,\psi_{C}\left(a\text{b},\epsilon \right)=\Pi_{a}\left(\psi_{C}\left(\text{b},\epsilon \right)\right). \end{array}$$

Per this property, Π_*a*_ is a function which given *any* starting sensory reading of the agent can determine the sensation it would experience after performing action *a* (provided the environment state stays constant during *a*). It must be reiterated that a crucial part is that this function operates on *sensory* data, which is precisely the only data available to the agent.

### 3.4. Prediction as an Internal Proxy of the Action Group

From there, let us now consider

(14) $$\begin{array}{l} \Pi :A_{C}\to \text{Bij}\left(S\right)\\ \;a\mapsto \Pi_{a} \end{array}$$

with Bij(S) the set of all bijections from S onto itself, i.e., Π maps abstract motor actions to their sensory prediction functions. As proven in Appendix 3 (see the Supplementary Materials), it establishes a group isomorphism between conservative actions $a\in A_{C}$ and their associated sensory prediction maps $\Pi_{a}\in \Pi \left(A_{C}\right)$, so that

(15) $$\begin{array}{l} A_{C}≅\Pi \left(A_{C}\right). \end{array}$$

While Equation (15) written as is might easily pass as benign, it is actually a very powerful result and the centerpiece of our argument. In a similar fashion to Equation (8) before it, this specifies how the algebraic structure of (conservative) actions —which largely governs the sensorimotor experience— appears as a contingency of the sensorimotor flow which can be picked up on by an agent as naive as outlined in section 3.1. Using the terminology introduced there, it shows how some *external* structures describing the interaction between agent and environment can be captured from the *internal* point of view. In turn, it is the enunciation —and the proof— of this result that motivate developing the formalism as in section 2, going as far back as absolute configurations **b** ∈ B and ambient space X. Equation (15) will be leveraged as part of the simulations in the following.

## 4. Simulating a 2D Version of Our Toy Model

Up until this point, the discussion has been kept to a purely theoretical level. The following section is now devoted to a simulated experiment illustrating the new proposed formalism. To this end it starts with a description of the experimental setup, highlighting how it manifests in the proposed formalism of section 2. Then, we describe what steps the agent goes through and how they relate to the theoretical results we put forth in the previous section. Finally, we review the observable results of these experiments to inspect how our earlier theoretical claims appear in practical cases.

### 4.1. Description of the Experimental Setup

**Figure 4:**
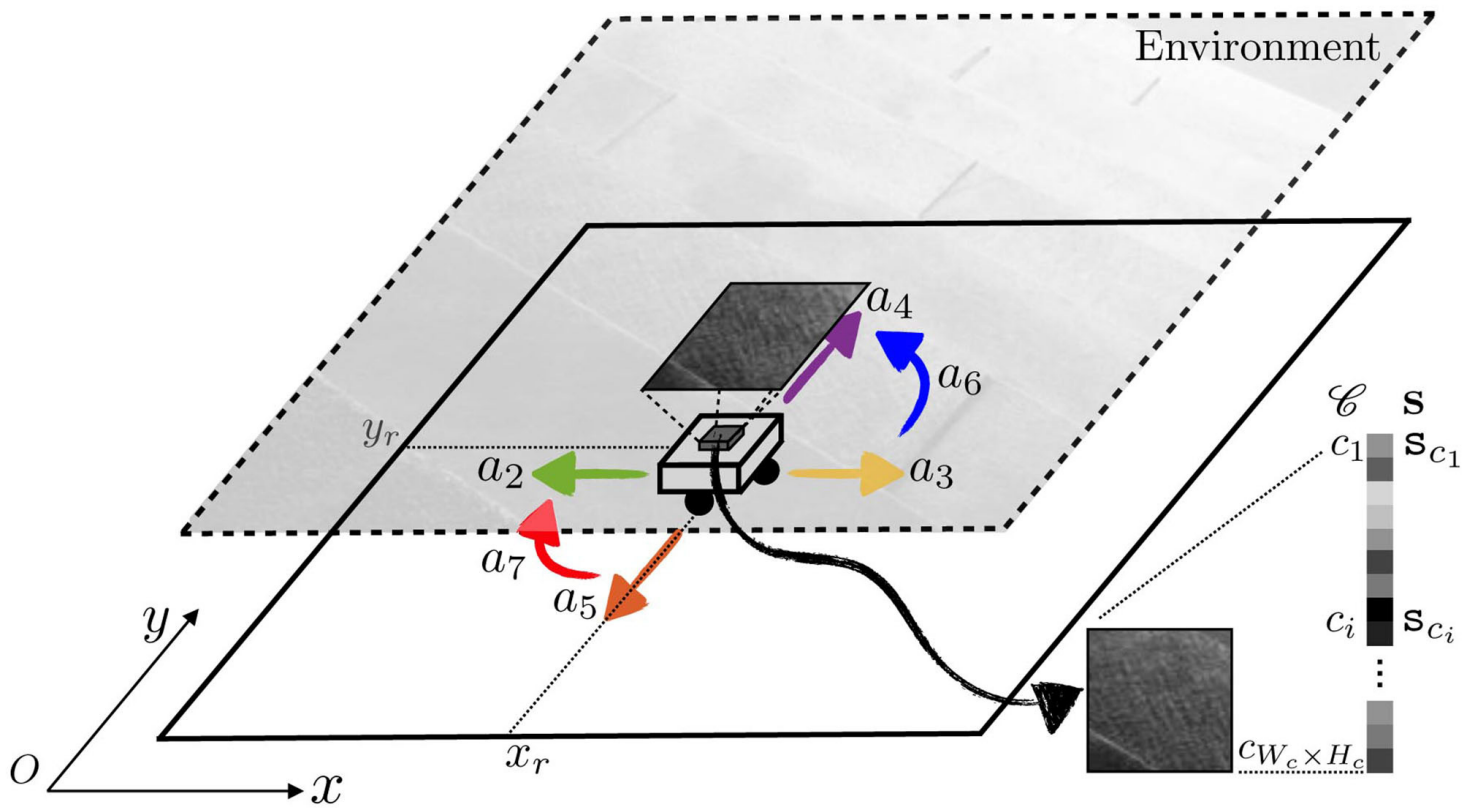
Experimental setup used in simulation to assess the proposed formalism. A holonomic agent is placed in a 2D environment which ceiling is made of a fixed grayscale image. The agent can move in this environment by applying seven different actions *a*_*k*_. A 10 × 10 camera pointed toward the ceiling is placed on the top of the agent and generates a sensory array **s** = (_s_*c*_*i*__)*i*_.

In the following, we will consider the 2D generalization of the illustrating case used in the previous sections. That is, the studied agent body is now made of a planar, rectangular camera sat atop omnidirectional wheels, see Figure 4. These allow for translations along both *x* and *y* coordinates, as well as rotations in the plane. The pixels of the camera are sensitive to the luminance of the ambient stimulus, which for our experimental purposes is a fixed grayscale image placed above the moving camera. Describing the problem in the terms of the developed formalism gives:

- the ambient space X is the plane ℝ^2^;
- the set of physical properties of space P is [0;255] the set of luminance values. Therefore, a state of the environment ϵ ∈ E is a function which takes points (*x, y*) of the ambient plane and map them to luminances as given by the data of the acquired image;
- the configuration space B is ${\mathbb{R}}^{2}\times S_{1}≅\;{\mathbb{R}}^{2}\times ]-\pi ;\pi ]$ to account for both position (*x, y*) and orientation θ of the robot on the plane;
- the sensory output of the agent is an array $\text{s}\in {\left[0;255\right]}^{W_{c}\times H_{c}}$, with *W*_*c*_ (resp. *H*_*c*_) the number of sensels/pixels in one row (resp. one column) of the camera. In the simulation, the image dimension is set to *W*_*c*_ = *H*_*c*_ = 10. Each of the components s_*c*_*i*__ of **s** are the sensory output of pixel *c*_*i*_, given by the luminance of the spatial location in the environment it is currently looking at. Importantly, the order of each pixel in **s** is chosen arbitrarily.

Let us define a set *A* of *seven* basic actions *a*_*k*_, *k* = 1, …, 7:

1. one identity action *a*_1_, mapping any current absolute configuration to itself;
2. four translations *a*_2_, *a*_3_, *a*_4_, *a*_5_, one for each direction of the basis axes on the plane, all of amplitude the size of 1 pixel. These are defined relative to the *current orientation of the agent*, which can end up distinct from external systems of axes when the agent rotates;
3. two 90° rotations *a*_6_, *a*_7_, to account for both clockwise and counter-clockwise turns.

These actions are depicted in Figure 4 with colored arrows. Note that the color convention used in this figure is the same used in the forthcoming figures for coherence.

Relative to the prior discussion about properties of motor actions, these are not strictly *conservative* as per the definition (9): indeed, consider *a*_5_ the elementary “forward” translation. While inner pixels of the camera will certainly exchange receptive fields, those in the front row will necessarily observe new areas of space after the agent has moved forward. Therefore, none of these front row pixels has any successor for *a*_5_, which precludes it from being strictly conservative. The same phenomenon of border impredictibility occurs for all translations, each with their respective side failing to verify the conservation property. We nevertheless proceed with the formalism on the basis that actions are at worst, informally speaking, “quasi” conservative. This is based on the quick analysis that, for a *N*-by-*N* square camera, this defect only occurs in *N* pixels which remains an order of magnitude fewer than the *N*^2^ total.

Representing the sensory configuration as numerical arrays makes the permutation of sensels into *N*_*c*_-by-*N*_*c*_ sparse matrices, where *N*_*c*_ = *W*_*c*_ × *H*_*c*_ is the number of sensels. Indeed, starting with any permutation ϕ:⟦1, *N*_*c*_⟧ → ⟦1, *N*_*c*_⟧ we can define a matrix *M*_ϕ_ ∈ *M*_*N*_*c*_, *N*_*c*__(ℝ) by

(16) $$\begin{array}{l} {M_{\phi }}_{i,j}=\{\begin{array}{cc} 1 & \text{iff }j=\phi \left(i\right),\\ 0 & \text{else}. \end{array} \end{array}$$

It can then be checked that for any array **s** = (_s_*i*_)*i* ∈ ⟦1, *N*_*c*_⟧_, the array **s**_ϕ_ = (_s_ϕ(*i*)_)*i* ∈ ⟦1, *N*_*c*_⟧_ obtained by permutating the components of **s** by ϕ verifies **s**_ϕ_ = *M*_ϕ_**s**. It is clear that working with such a representation incurs a large memory overhead (with only *N*_*c*_ of all $N_{c}^{2}$ coefficients being non-null). Furthermore, finding a permutation is known to be a problem of exponential complexity. However, we do not aim to propose a scalable implementation in the following, but rather to illustrate as a proof of concepts the developments in section 3.

### 4.2. Description of the Experiments

The proposed simulation can be decomposed as a sequence of two related, successive, experiments. First, these are briefly described in a global manner so as to go through the flow of the experiment. Then, each experiment is described in greater detail with respect to its implementation. It is in this second part that relevant proofs ensuring both completion and correctness of the endeavor are provided. In this setup, the robot is given a set $A_{\text{init}}$ of $n_{A}$ unknown actions drawn in the set of *combinations* of actions of *A*. Although *A* was designed for convenience from an external point of view, $A_{\text{init}}$ may not accurately reflect it. Indeed, for random draws there is a high likelihood of missing actions when $n_{A}$ is small, of duplicate actions when it is large. However, as discussed previously these notions do not yet make sense to the agent, which can only “run” actions drawn. Importantly, at first the considerations will be restricted to the case where $A_{\text{init}}=A$. This is a possibly strong assumption about the initial fitness of readily available commands to the “objective” capabilities of the agent. The influence of this choice and the effect of less optimally designed starting command shall be discussed in the final part of this section.

The first part of the experiment is one of *motor babbling*. During it, the agent effectively runs its available actions $a_{k}\in A_{\text{init}}$ multiple times and tries to figure out whether they are *conservative* by computing their associated sensel permutation map. This is realized as a sequential process: at timestep *t*_*n*_, the agent chooses and runs an action $a_{k}=a\left[t_{n}\right]\in A_{\text{init}}$, and the absolute configuration **b**[*t*_*n*_] = (*x*[*t*_*n*_], *y*[*t*_*n*_], θ[*t*_*n*_]) is accordingly changed to **b**[*t*_*n*+1_] = *a*_*k*_**b**[*t*_*n*_]. Corresponding sensory array **s**[*t*_*n*+1_] = (_s_*i*_[*t*_*n*+1_])*i*_ is then used to proceed in the computation of the (candidate) permutation matrix *M*_*a*_*k*__ of *a*_*k*_, with the details of the update rule discussed in the following subsection. It must again be stressed that we do not consider the actual time *t*_*n*+1_−*t*_*n*_ required to perform the action *a* as a relevant information of the proposed sensorimotor framework. It may vary for distinct actions without it affecting whatsoever the sequence of experienced absolute configurations **b**. During this exploration, the state of the environment ϵ is also allowed to vary with time so long as it is not updated during the generation of *a*_*k*_, i.e., ϵ can change *between* actions. This is achieved during the simulation by entirely changing the grayscale image presented to the agent between each action. In the following, the choice of action is randomly made at each timestep. This may at times slow the learning of the permutation matrices and could certainly be improved, for instance by introducing a necessarily intrinsic criterion like curiosity as in Oudeyer et al. (2005). However, the specific case studied here is simple enough that a random strategy suffices to obtain good results.

Once this first step is complete, the agent computes all products of (quasi) permutation matrices to make the resulting set of matrices. As per Equation (4), this set is precisely the one of all matrices that decompose over the $M_{a_{k}},a_{k}\in A_{\text{init}}$. Following our argument about the groups of prediction functions and motor actions being isomorphic, this set can be taken as the *global* understanding of its motor capabilities the agent has acquired. Here “global” denotes that new structure, absent from the first empirical phase which was limited to $A_{\text{init}}$, emerged from the computation of products. Finally, the effect of changing the set of actions available at start on the structure graph discovered in the second experiment is studied in a third part (see section 4.3.3).

#### 4.2.1. Learning the Prediction Through Sensorimotor Interaction

The first experiment performed by the agent is computing, where possible, the permutation matrix associated to each of its available motor actions. This is done according to the following procedure: at the beginning of the sensorimotor experience, to each starting action $a_{k}\in A_{\text{init}}$ associate a *N*_*c*_ × *N*_*c*_ matrix *M*_*a*_*k*__ where *N*_*c*_ is associated the number of sensels. This matrix is initialized so that all of its coefficients are 1. Then, at the end of timestep *t*_*n*_ where it performed action *a*_*k*_ (that is *a*[*t*_*n*_] = *a*_*k*_), the agent uses its sensory output arrays both previous (**s**[*t*_*n*_]) and current (**s**[*t*_*n*+1_]) as per the update rule:

(17) $$\begin{array}{l} {\left(M_{a_{k}}\left[t_{n+1}\right]\right)}_{i,j}=\{\begin{array}{cc} 1 & \text{iff }{\text{s}}_{j}\left[t_{n+1}\right]={\text{s}}_{i}\left[t_{n}\right]\text{ and }{\left(M_{a_{k}}\left[t_{n}\right]\right)}_{i,j}=1\\ 0 & \text{else}. \end{array} \end{array}$$

Let us first observe that in this rule the only possible change in coefficients is going from 1 to 0: whenever a coefficient (_*M*_*a*_*k*__[*t*_*n*_])*i, j*_ is already 0, the condition of the first case automatically fails so that its value stays at 0. Therefore, the rough dynamics of the update is that while all coefficients start at 1, some are eventually switched to 0 upon exploration until matrices converge to a final (possibly null) form.

One can note that this is a very drastic choice compared to the more usual soft incremental rules. This offers increased simplicity, such as in Appendix 4 (see the Supplementaty Materials) where an argument is provided that for any conservative motor action this algorithm makes the empirical matrix *M*_*a*_*k*__ converge to the associated permutation matrix *M*_σ__*a*__*k*___. Moreover, we argue that obtaining said convergence with such an unforgiving rule is strong evidence toward the systematic, rather than statistical, nature of the supporting mechanism. The argument also proves that for non-conservative actions, under the same richness hypothesis the associated empirical matrix will converge to the null matrix. This fact allows the robot to naively distinguish between conservative and non-conservative actions, should he be given the capability to perform both on startup.

#### 4.2.2. Inferring Motor Structure From Learned Interaction

In the second phase of the experiment, the agent uses the prediction functions it discovered for elementary conservative moves to infer how combinations of these moves relate to each other. Indeed, it was proved in the previous part that for any conservative actions *a* and *a*′ with associated permutation matrices *M*_σ_*a*__ and $M_{\sigma_{a^{\prime }}}$, it is true that

(18) $$\begin{array}{l} M_{\sigma_{a^{\prime }}}M_{\sigma_{a}}=M_{\sigma_{a^{\prime }a}}. \end{array}$$

In the case of actions which are not strictly conservative, such as those in the simulation, equality in the previous equation is not guaranteed. This happens because in the $M_{\sigma_{a^{\prime }}}M_{\sigma_{a}}$ expression, all the loss of information of *a* and *a*′ on their respective boundaries is accumulated, whereas *a*′*a* might recoup some of it, e.g., when *a*′ = *a*^−1^. However, multiple expressions should at least yield non-contradictory prediction, that is whenever one specifies a pair *c*_*i*_→*c*_*j*_ another other cannot assert *c*_*i*_→*c*_*k*_ with *j*≠*k*. As long as these combinations are kept short enough to limit the accumulation, this non-contradiction criterion can be used by the agent to internally infer the sensory prediction of *any* combination of the moves it empirically learned. This is used in a Dijsktra-like process to build a graph of prediction matrices, which runs as follows, see Algorithm 1: starting from a prediction matrix *M*_0_ corresponding to any origin action *a*_0_, each of the known matrices $M_{a_{k}},\;a_{k}\in A_{\text{init}}$ are applied to yield both a set of new neighboring “end points” $N_{M_{0}}:=\left\{M_{a_{k}}M_{0},\;a_{k}\in A_{\text{init}}\right\}$ and for each pair (*M*_0_, *M*_*a*_*k*__*M*_0_) a directed edge *M*_*a*_*k*__. This is then recursively applied to all newly discovered end points, while those that were previously visited (as the prediction matrices *can* be compared for equality) are discarded. However, the resulting graph would in most cases be infinite, therefore a stopping rule must be chosen. In our case, we chose to explore up to a given depth parameter in graph edge distance.

**Algorithm 1:** Dijkstra-like algorithm for live construction of action group graph.

Input
A The set of all matrices learned in exp. 1
D A bound on length of matrix combinations used
O A reference matrix around which to explore
**Output**
G A local view of the combinatorial graph of matrix products around O, using edges in A

Add O to collection U
O.depth ← 0
Add node O to G
while U is not empty **do** ⊳ True iff the neighborhood of some node K is still Unexplored
K ← node in U
for all *M*_*a*_ in A do⊳ Test all learned predictions starting from node K
P ←*M*_*a*_*K*
P.depth ← K.depth + 1
if P.depth ≤ D then
B ← False
for all node C in G do ⊳ Test previously discovered nodes for equality
if predictions for P and C match then
B ← True
Set edge *M*_*a*_: K → P in G
end if
end for ⊳ END for all node C in G
if B is False then ⊳ Branch taken iff P := *M*_*a*_*K* was not previously discovered
Add P to U
Add node P to G
Set edge *M*_*a*_: K → P in G
end if
end if ⊳ END if P.depth ≤ D
end for ⊳ END for all *a* in A
Remove K from U
end while ⊳ END while U is not empty

### 4.3. Results

This subsection is devoted to the evaluation in simulation of the previous points, divided in three successive experiments. The first one illustrates how the agent can build the permutation matrices associated to each of its conservative actions; a discussion about the convergence and the statistics of this experiment is then proposed. The second one exploits the permutation matrices just obtained to structure its own actions through a graph of their combinations; a discussion about its fidelity as a representation of the action group A is proposed. In the third and final one, the effect of varying starting action sets on the structure discovered is studied, concluding the subsection.

#### 4.3.1. Experiment 1: Discovering the Permutations

##### 4.3.1.1. Building the permutation matrices

To begin with, the simulated robot in Figure 4 is placed at a random 2D position inside the image to be explored. The available action set is defined as $A_{\text{init}}=A$ so that $n_{A}=7$. Then, at each time step *t*_*n*_, a random action $a_{k}=a\left[t_{n}\right]\in A_{\text{init}}$ is run, and the associated permutation matrix *M*_*a*_*k*__ is updated according to (17). After this update, the agent is able to evaluate if these matrices have finished converging and therefore can decide when to stop the exploration. An entropy-like internal criterion is proposed to quantify this convergence, along

(19) $$\begin{array}{l} \;C\left(M\right)=1-\frac{1}{N_{c}{\text{ log }}_{2}\left(N_{c}\right)}\overset{N_{c}}{\underset{i=1}{\sum }}H_{i},\\ \text{where }H_{i}=-\overset{N_{c}}{\underset{j=1}{\sum }}\frac{M_{i,j}}{\mu_{i}}{\text{ log }}_{2}\left(\frac{M_{i,j}}{\mu_{i}}\right),\\ \text{ and }\mu_{i}=\frac{1}{\text{max}\left(1,\sum_{j=1}^{N_{c}}M_{i,j}\right)}. \end{array}$$

In this criterion, *H*_*i*_ is the entropy of the post-action output of sensel *c*_*i*_ as a random variable of the pre-action outputs of all sensels *c*_*j*_. Therefore, it measures which degree of surprise remains in the determination of which (if any) sensel is successor to *c*_*i*_. Finally, this makes *C* into an average measure of certainty in the discovery of successor sensel pairs, going in non-decreasing trajectories from 0 at initialization to 1 at permutation matrices. Consequently when it obtains the updated matrices $M_{a_{k}}\left[t_{n+1}\right],\;a_{k}\in A_{\text{init}}$ the agent computes all *C*_*k*_[*t*_*n*+1_] = *C*(*M*_*a*_*k*__[*t*_*n*+1_]) to assess the state of its discovery, stopping its exploration when all the *C*_*k*_ have reached 1.

**Figure 5:**
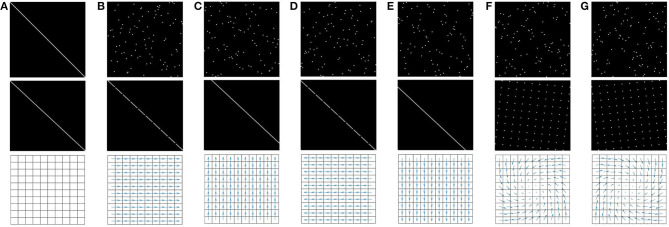
Representation of the seven binary 10^2^ × 10^2^ permutation matrices *M*_*a*_*k*__ corresponding to the actions *a*_*k*_ possibly generated by the agent, where a 0 (resp. a 1) is represented as black (resp. white). (First row) Matrices obtained for a random organization of the sensels outputs *s*_*i*_ inside the sensory array **s**. (Second row) Matrices obtained for a well-chosen sensels arrangement, where each pixel values are stored line by line in **s**. (Third row) Interpretation of the permutation matrices (either from the first or second row) directly on the physical 10 × 10 pixel array: if a 1 is present at line *i* and column *j* of a matrix *M*_*a*_*k*__, then an arrow joining pixel *i* to *j* is plotted. Note that the arrow length has been resized for actions 6 and 7 (i.e., rotations) to enhance readability. **(A)** *M*_*a*_1__. **(B)** *M*_*a*_2__. **(C)** *M*_*a*_3__. **(D)** *M*_*a*_4__. **(E)** *M*_*a*_5__. **(F)** *M*_*a*_6__. **(G)** *M*_*a*_7__.

After convergence, the resulting matrices for all seven actions shown in Figure 4 are depicted in Figure 5. In this figure, a 0 (resp. 1) is represented in black (resp. white). Since the agent has no knowledge of its sensor geometry, the position of its sensels (i.e., pixels) inside the sensory array **s** (i.e., the flattened image) is randomly chosen. In this case, the resulting permutation matrices for each action is depicted in Figure 5 (top), demonstrating the fact that those matrices are not easy to understand from an external point of view. If one now selects a more natural ordering of the pixels inside **s**, like a line by line arrangement, one then gets the permutation matrices in Figure 5 (middle). With such an arrangement, an external observer is now able to get a clearer intuition about the effects of each action on the pixels permutations. Nevertheless, these two different sets of matrices, as two contingent images of the same underlying structure, are purely equivalent from an internal point of view. This can be illustrated by mapping the permutation on the overall sensor to better catch how the agent has been able to discover the underlying spatial transfer between sensels. This is done by plotting the sensel pairs along which values are transferred as proposed in Figure 5 (bottom). In this figure, the 10 × 10 pixel grid of the simulated camera is represented together with arrows connecting each sensel to its successor. While such a representation requires external knowledge in the sensor geometry, the arrows are entirely determined by the internal permutation matrices from either of the two sets presented. It is thus a convenient external way to display that each matrix has actually captured the pixel shift induced by each action. For instance, with such a visualization, it is now very clear that (*a*_2_, *a*_3_), (*a*_4_, *a*_5_), or even (*a*_6_, *a*_7_) are all found to be pairs of inverse actions; this specific capability will actually be exploited in section 4.3.2 to structure the agent set of actions.

##### 4.3.1.2. A discussion about the dynamics of convergence

**Figure 6:**
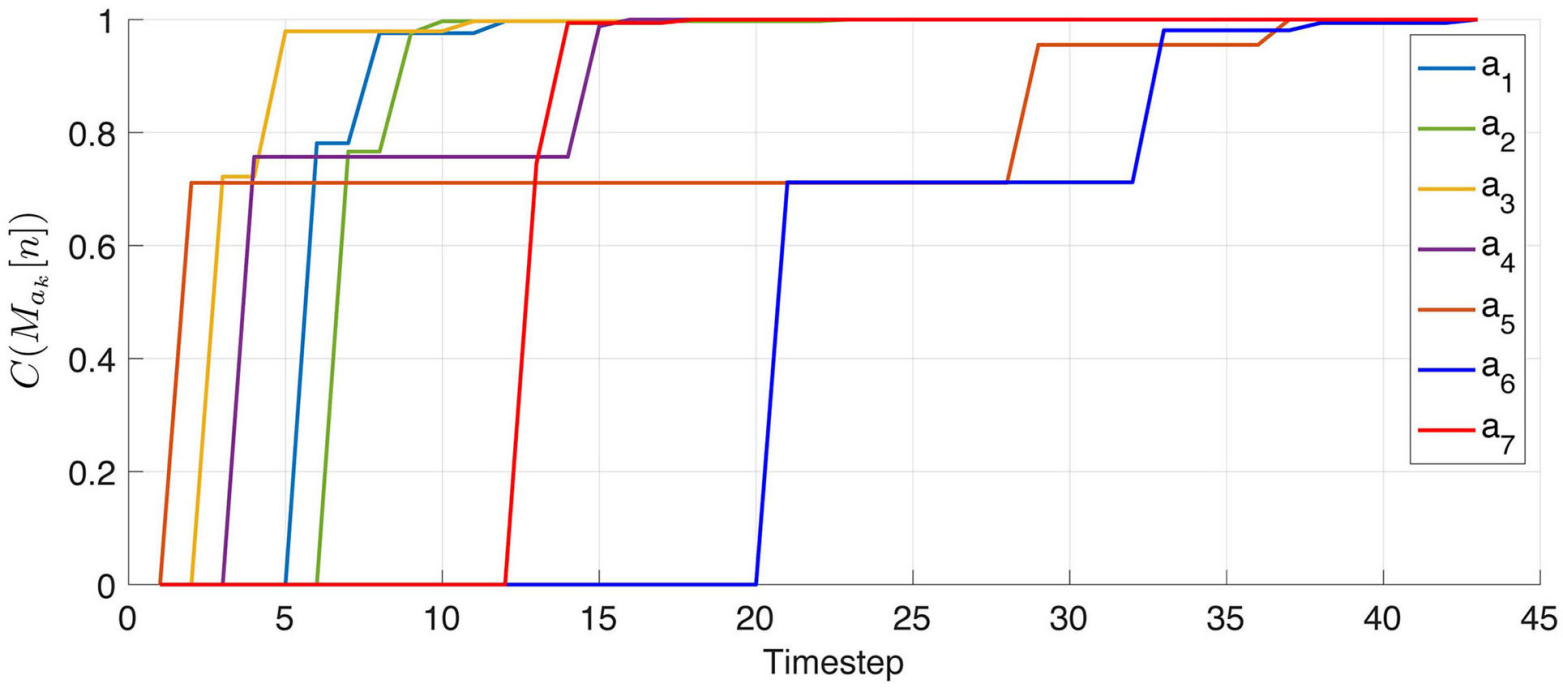
Representation of the criterion *C*_*k*_ = *C*(_*M*_*a*_*k*_) for the seven actions *a*_*k*_. Each jump in this figure corresponds to a reevaluation of the criterion happening at a timestep when the corresponding action has been drawn in the set in $A_{init}$. As expected, the criterion starts from 0 to reach 1, indicating that all possible permutations have been found.

It is clear from Figure 5 that at some point the agent captured the permutation to the best of its capabilities. One therefore proposes to study the dynamics of the convergence of the approach w.r.t. the experimental time step *t*_*n*_. For the remainder of experiment 1, we now keep the image constant during all the simulation, so as to better assess the influence of the experienced environment on the results. First, the internal criterion *C*_*k*_ = (*M*_*a*_*k*__) defined in Equation (19) is evaluated at each *t*_*n*_ and each *a*_*k*_, resulting in the plot in Figure 6. One can then confirm that the *C*_*k*_ increase from 0 (all elements in the matrices are initialized at 1) to 1 (all successor pairs have been discovered). It also appears that for each particular action *a*_*k*_, the associated criterion increases in sparse jumps because its matrix *M*_*a*_*k*__ is only actually updated at the random time steps when *a*_*k*_ is drawn. Figure 6 also illustrates the fact that the amplitude of these jumps decreases over the experiment. For the starting conditions of this experiment, a detailed analysis shows that about seven realizations of each action are necessary to fully discover the target permutation matrices. But it also appears that most of the initial 1s in the matrices are wiped out very early, with a criterion value *C*_*k*_[*t*_*n*_] ≈ 0.7 after only one execution of the corresponding action *a*_*k*_. However, one still questions whether the differences in the dynamic of all actions is a random occurrence of this particular exploration, or there is an intrinsic variance in difficulty in learning between actions.

##### 4.3.1.3. A statistical analysis about richness of the environment

**Figure 7:**
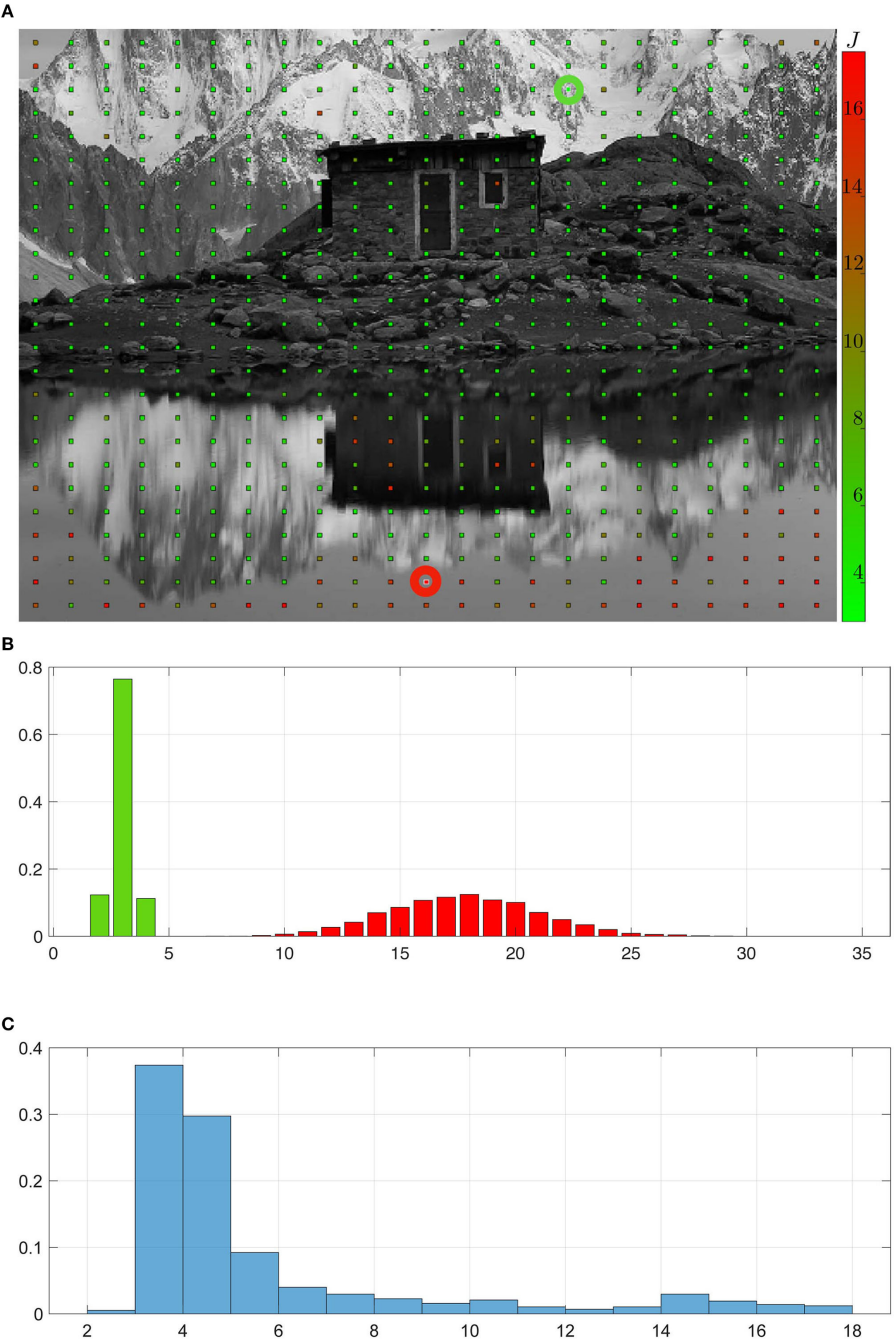
Statistical analysis of the permutation matrices building process. **(A)** Environment explored by the agent. Each point in this environment corresponds to a starting position around which the agent draws actions to build the permutation matrices *M*_*a*_*k*__. Counting the mean number of jumps in the criterion curves *C*_*k*_ for each realization of the exploration around a given starting point and each action leads to the value *J* representing the difficulty to build the corresponding matrices. A high (resp. low) J value in red (resp. in green) corresponds to areas in the environment harder (resp. easier) to exploit for sensory prediction. **(B,C)** Normalized histograms of the number of jumps in the criterion curves *C*_*k*_ averaged across actions. **(B)** Focus on the histograms obtained around two different starting conditions corresponding to low (resp. high) *J* value highlighted by a green (resp. red) circle in the environment. **(C)** Overall normalized histogram for all actions and all starting positions in the environment, showing that most of the permutation matrices are correctly obtained after a low number of action run.

The answer to the previous question can be obtained by performing an empirical survey (i) by averaging over random explorations for given starting conditions, and (ii) by varying these starting conditions and comparing the resulting performances. With such a study, (i) will allow to quantify the influence of the randomness in exploration, while (ii) lets us assess how the properties of the environment influence the discovery of permutations. For this experiment, the environment is made of the image shown in Figure 7A, where the starting points of each exploration is depicted as a grid of points on it. At each of these points, 1,000 random explorations are conducted, each of them consisting in a random run of actions *a*_*k*_ as in section 4.3.1.1, resulting in 1,000 sets of seven *C*_*k*_ curves as in Figure 6. For each random exploration *l* and each action *a*_*k*_, the number of jumps *J*_*l*,_*a*__*k*__ in the *C*_*k*_ curve obtained is taken as a measure of difficulty in learning the permutation. The average $J=\frac{1}{L}\underset{l}{\sum }\underset{k}{\sum }J_{l,a_{k}}$ of *J*_*l*,_*a*__*k*__ over all actions *a*_*k*_ and explorations *l* at a given starting position is depicted as the color of the grid in Figure 7A, with *L* = 1,000 (runs) × 7 (actions). Green points correspond to a low number of jumps *J*, while red ones are representing higher values. One can observe that the points are overwhelmingly green, and that the red ones are restricted to precise areas in the picture. These correspond to areas with locally low contrast, such as the sky (in the top left corner) or its reflection (in the bottom). The extremal conditions corresponding to the two green and red highlighted points are further compared. For each of them, the distribution of the *J*_*l*,_*a*__*k*__ is plotted as an histogram in Figure 7B. Clearly, green points correspond to areas in the environment where the permutation matrices can be discovered in at most five executions of actions. On the contrary, at red points the agent must wait for about 17 on average, and up to 35, executions before it has obtained the same results. This illustrates how the richness of the environment might influence the agent ability to capture the structure of its sensory prediction. On a more global scale, Figure 7C shows the distribution of the *J*_*l*,_*a*__*k*__ for all random explorations, indiscriminately of the starting position. This corroborates the observation that most positions in the image are green, i.e., lead to easy convergence. It appears that for a randomly selected starting position, there is more than 66% of chance of permutation matrices being discovered in <4 executions of their corresponding actions.

#### 4.3.2. Experiment 2: Structuring Actions by Combination

From the previous experiment, we now have as many permutation matrices *M*_*a*_*k*__ as we have actions in $A_{\text{init}}$. As outlined in section 4.2.2, one can then use them to build a *graph of prediction matrices* by following Algorithm 1. Recall that in this graph, a node is a permutation matrix obtained as a combination of the *M*_*a*_*k*__ matrices, while there is a *M*_*a*_*k*__ edge from matrix *M* to *M*′ iff $M^{\prime }=M_{a_{k}}M$. Therefore, all edges in the graph correspond to the permutation matrices built during experiment 1. According to Equation (15), this graph is isomorphic to the graph of corresponding actions, meaning all properties discovered of any combination of matrices holds true for the corresponding combination of actions. As an example, if one discovers that *M*_*a*_1__ = *M*_*a*_2__*M*_*a*_3__, then one also has *a*_1_ = *a*_2_*a*_3_.

**Figure 8:**
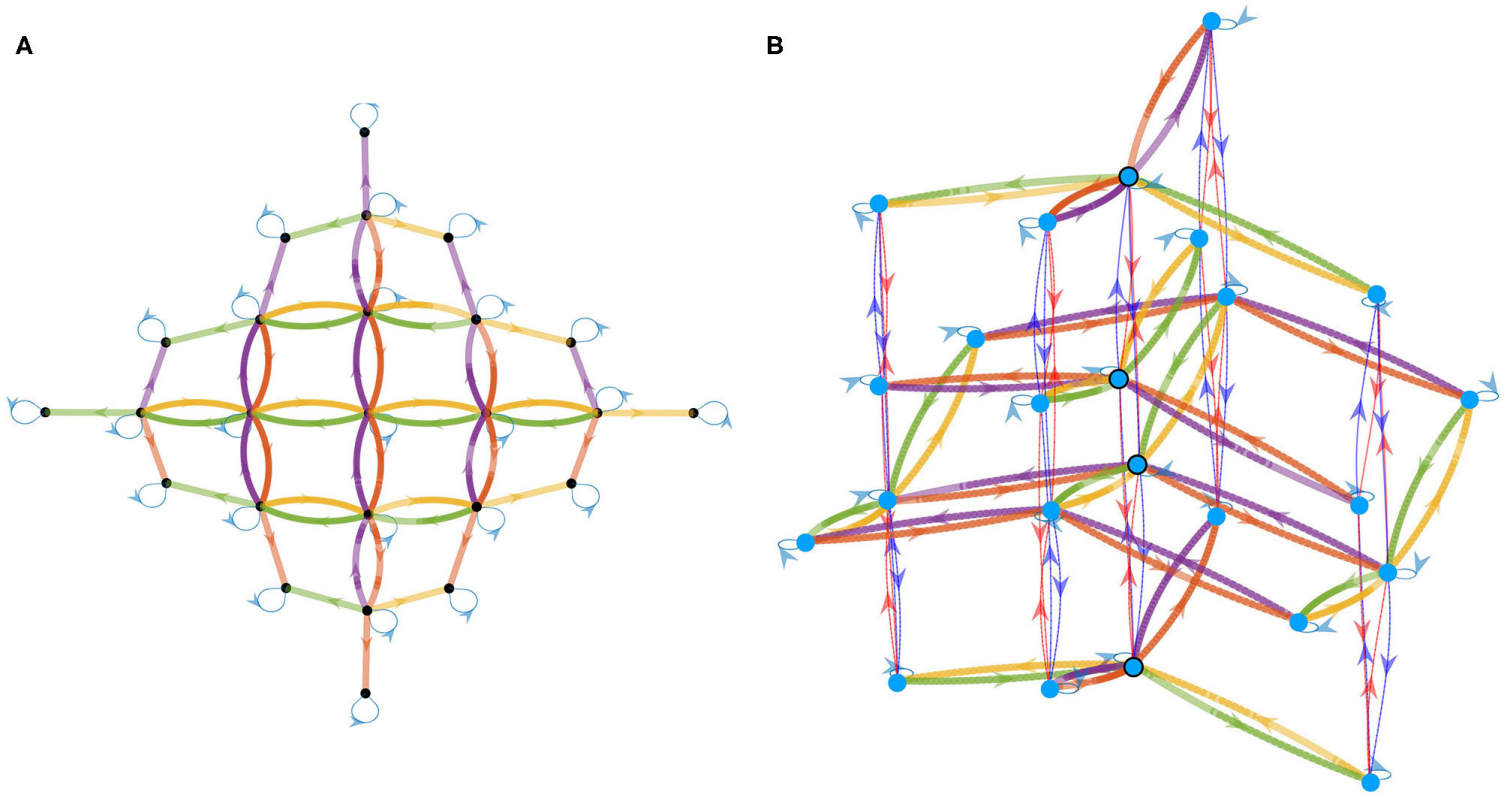
Directed graph of permutation matrices Mak -and thus also of corresponding actions ak as per Equation (15)– obtained by combination of these matrices. Color conventions for edges match the color of each action in Figure 4. The depth up to which new nodes are explored has been limited for visualization purposes by setting low depth parameters in Algorithm 1. **(A)** Graph obtained when considering only the actions corresponding to translations of the agent in the environment. **(B)** Graph obtained when considering all actions: the 2D graph from **(A)** is now enriched with a third dimension supporting the changes of orientation induced by the agent rotations in the environment.

As a first step, let us consider only the actions corresponding to translations in the environment, i.e., *a*_6_ and *a*_7_ are discarded from $A_{\text{init}}$. This *a priori* selection is only made to simplify the visualization of the graph at first. After applying Algorithm 1 to the matrices shown in Figure 5, one gets the directed graph in Figure 8A, where all the color conventions are consistent with experiment 1. This particular graph has been built for a maximum depth set to 3 and with *M*_*a*_1__ taken as the origin of the graph. Note that the depth of this graph has been maintained voluntarily low so as to help in the reading of the graph. Note also that the arbitrary choice of origin makes all of its neighbors themselves correspond to one of the *M*_*a*_*k*__ discovered in experiment 1 since they all occurred as *M*_*a*_*k*__*M*_*a*_1__ = *M*_*a*_*k*__ products, whereas all other nodes are indeed new matrices.

This graph mirrors many algebraic properties of the *M*_*a*_*k*__ as captured by the internal experience. Indeed one can first observe that the light blue arrow leads from any given node *M* to itself, which corresponds to *M*_*a*_1__ being the identity matrix *I*_*N*_*c*__. Furthermore, one can note that the graph obtained is, up to its borders, completely homogeneous; that is the neighborhoods of each interior nodes share the same geometry. This even extends to the color of edges matching, so that some of them form pairs. One can for example verify that whenever a yellow edge goes from node *M* to *M*′, there is a green edge from *M*′ to *M* and no other one. This identifies the corresponding actions to be inverses w.r.t. successive execution since from any starting node, taking first the green (resp. yellow) edge then the yellow (resp. green) one forms a loop. The same can be said of the red and purple colors, which are found to correspond to another pair of inverse actions. At last, the four central squares correspond to the commutativity of the *a*_*k*_ used: indeed one can see on the graph that taking the red edge first, then the green one always leads to the same node as green first, red second.

While those observations were discussed as properties of the permutation matrices, the actual result is their representing properties of the abstract motor actions *a*_*k*_. And indeed one can check that the blue arrow corresponds to the identity action *a*_1_, that the inverse pairs (yellow, green) and (orange, purple), respectively correspond to (rightward, leftward) and (forward,backward) translations, and that the commutativity discussed is that of “forward then left” being the same as “left then forward.” While these facts seem obvious from an external point of view, they were not part of the initial knowledge of the agent discussed in section 3.1. This only appears as a consequence of the agent capability to predict the sensory consequences of its own actions built during experiment 1. On a functional level, this is very similar to the property of motor sequence compression exhibited by RNNs performing sensorimotor prediction in Ortiz and Laflaquière (2018); in fact we argue that it is the same phenomenon that is picked up on by the neural networks and that it is intrinsically related to sensorimotor prediction as developed in section 3.

This also applies to the graph shown in Figure 8B obtained when considering all seven actions, i.e., the two rotations corresponding to actions *a*_6_ and *a*_7_ are now included in the analysis. This plot, obtained through a classical force-directed algorithm, shows the same 2D graph of translations obtained before, but enriched with a third dimension supporting the change of orientation induced by rotations. Again, the depth of the graph is maintained low to keep things legible. The global structure of the graph can be described as a disjunction of 2D subgraphs corresponding to translations at fixed orientation. Each subgraph is therefore equivalent to each other up to a rotation as can be seen by the edges colors shifting between the planes. As an example, one can see that the same node in the graph can be reached by following either (green, dark blue) and (dark blue, purple), or (left, turn left), and (turn left, forward) in terms of actions seen from an external point of view. Figure 8B also shows that rotations are limited to the third vertical dimension in which they form cycles at constant position in the planar subgraph. This property is highlighted in the graph by the four circled nodes which figure the same agent position for the four possible orientations. The cycle simply mirrors the external observation that taking four π/2 rotations successively takes one back to the initial orientation. Importantly, this could constitute an internal signature of rotations as opposed to translations.

In the end of this second experiment, the agent has thus been able to discover a structure of its actual group of motor actions. The agent now has access to algebraic relations between its own actions which relate to its motor capabilities. This knowledge also allows it to generalize the sensory prediction it discovered in experiment 1 to all the combinations considered in the graph. Nevertheless, one has to keep in mind that all the actions considered in these experiments are not exactly conservative in the sense of Equation (9). Indeed, they fail at conserving spatial information on the border of the simulated camera. However, results show that conservativity holding true for all internal pixels allows for discovering the aforementioned properties. Because all calculations are made on actual current outputs of the pixels, one obvious consequence is that the agent has no way to predict what happens outside of its field of view, and so far it keeps no memory of it. Therefore, the results only hold for a very local movement w.r.t. the dimension of the agent. However, the discovered structure stays true whatever the initial position of the exploration.

#### 4.3.3. Experiment 3: Exploiting the Graph to Improve the Representation of the Action Set

In the previous results, we have run the experiments with an experimental starting action set $A_{\text{init}}$ conveniently set to *A*, the set of externally defined movements. While this has been useful at first to yield easily recognized structure in Experiment 2, it is a crucial point that the results do not depend on this strong assumption. Therefore, the same two part experiment is conducted with the difference that the starting action set $A_{\text{init}}$ the agent can run is not arbitrarily set to *A* anymore. Instead, it is now drawn in the set of *combinations of* actions *a*_*k*_ ∈ *A*. Three important cases are now possible : first, it may be that some of the *a*_*k*_ are “missing” in $A_{\text{init}}$; on the contrary, duplicates may have been drawn so that the agent can run $a,a^{\prime }\in A_{\text{init}}$ which are effectively the same action (i.e., ∀**b** ∈ B, *a***b** = *a*′**b**). Finally, it may even have drawn “complex” actions *a*∉*A*, that is actions that can only be obtained by combining some of the *a*_*k*_.

**Figure 9:**
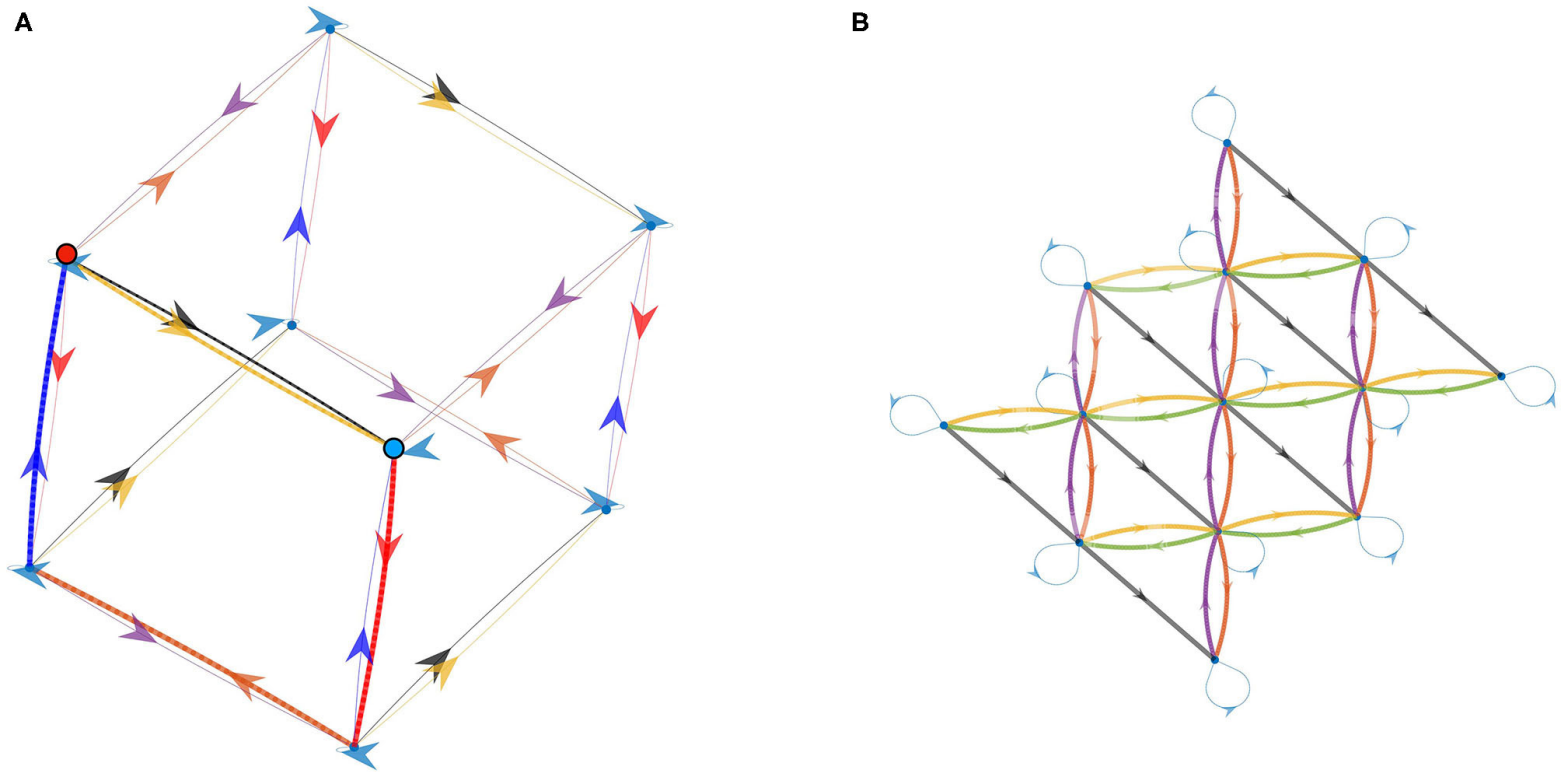
Illustration of the effect of drawing starting actions at random on the discovered structure. **(A)** Graph obtained with a duplicate starting action (depicted in black) and a missing action (*a*_2_, green in other figures). A path made of 3 edges (red, orange, blue) equivalent to the missing starting action is highlighted, providing an inverse to the (also highlighted) yellow edge. **(B)** Graph obtained by adding a combination (depicted in black) to the set of starting actions. Here, the action set was limited to translations to keep a clear visual.

The three situations are illustrated in Figure 9, where the graphs obtained at the end of Experiment 2 are drawn for various starting action sets. In both cases, both the complexity of the starting action set and the depth of the depicted graph have been limited to keep the discussed structure as readable as possible. Figure 9A depicts the first two cases: the agent was given a duplicate action from *A* as well as missing one. This can be assessed in the resulting prediction graph by the yellow and black arrows which relate the same ordered pairs of nodes, e.g., from the highlighted red to blue nodes, and the lack of an inverse arrow that would match them. Note that while the absence of this inverse (green) arrow represents the lack of a “direct” inverse action *in* $A_{\text{init}}$, the emerging structure from the graph allows for the determination of an inverse *path* as highlighted by the bold (red, orange, blue) arrows. From an external point of view, this basically means that if the agent has no action to translate itself to its left, it can instead rotate clockwise, then move backward, and finally rotate counter-clockwise to reach the correct orientation. Interestingly, this phenomenon where a missing inverse can be otherwise obtained by combination of other actions can only occur when the agent is able to rotate. The third situation is depicted in Figure 9B, where the experiment was conducted with the robot given the additional action *a*_8_ “forward then rightward” along *a*_8_ = *a*_5_*a*_3_ in addition to the translations of *A*. The choice not to give the agent its defined rotations *a*_6_ and *a*_7_ serves only to get an easily legible picture of the resulting graph, much like in Figure 8A, and does not impact the following. This additional move *a*_8_, which we as an external observer know to be a combination, is studied like all other basic actions by the agent during the motor babbling phase. It means that the agent has no cue about *a*_8_ being an actual combination of two other actions. In the end of the experiment, the obtained graph of action exhibits this additional action *a*_8_ as black edges, as shown in Figure 9B. From this graph, one can easily see that, from any point, it is indeed equivalent to follow either the black arrow or first the orange and then the yellow ones. The agent has thus been able to discover the action combination property.

These graphs therefore show two important results. The first conceptual comment is that the validity of the proposed experiment is not conditional to a perfect match between ideal, “objective” moves of the agent and actions it is effectively able to perform at start. This is a desirable property for genericity and our goal of bootstrapping, for it allows to avoid justifying said match. The second, more practical, comment is that the graph resulting from the experiment can be used by the agent to select an action set “better” than $A_{\text{init}}$. Indeed, the redundancy between edges (or paths of edges) in Figures 9A,B represent the agent discovering it can discard the actions corresponding to black edges *without losing capabilities*, i.e., while keeping all nodes reachable. It can then be used to prune the available action set to a *minimal* set generating the same group, in the sense of Equation (4). Determining a criterion for selecting which actions are kept and which are discarded could functionally correspond to a basis for invariant principles in motor actions of the agent (Flash and Hogan, 1998). The agent may also *expand* its action set with new actions that verify useful properties: for example, in the case depicted in Figure 9A it can package the bolded path of edges into a single action so that it gets a missing inverse.

## 5. Conclusion

This paper was devoted to the introduction of a variational extension of a previous framework into the sensorimotor framework, extending the scope of such approaches to naive agents able to move freely in their environment. We demonstrated how, despite their extremely limited starting capabilities, these agents could exploit said framework not only to perform sensory prediction, but also to structure their own actions. The proposed formalism has been assessed in simulation as a proof of concept, with a naive agent able to (i) build for each of its actions some permutation matrices associated to its own sensory array, and (ii) exploit them to structure its own set of actions. These experiments were conducted here in a somewhat simplistic experimental setting to keep the simulated situation as close as possible to the theoretical exposition. However, their transparent exploitation of the formal mechanisms we explicitly isolated yields valuable insight as to similar results, which related works otherwise achieved in more realistic conditions.

Implementing a formal version of sensory prediction comes with many interesting perspectives, as it was shown to yield crucial properties both in the original cognitive psychology literature and in the previous robotic contexts. We hypothesize that it can be used to better understand the emergence and properties of capabilities often related to that of sensory prediction, both from robotics and from cognitive sciences, such as those mentioned in the Introduction. These include, e.g., motor control, motor planning, isolating proprioception, suppression of self-induced changes or object perception. These capabilities therefore constitute potential applications to which further study could be devoted from there.

Nevertheless, the applicability of the proposed paradigm to real agents or robots is still an opened question. First, it is clear that most of the actions an agent will be dealing with are not strictly conservative, but rather *quasi*-conservative like in the simulations conducted in this paper. While not extensively studied in this paper, some ongoing mathematical developments show that their properties still allow to reach the same concepts of sensory prediction and action structuration. Then, the fact that the sensory prediction relies on exact sensory values shifts inside the non noisy sensory array is not very realistic. Introducing stochastic matrices instead of permutation ones constitutes a promising way to deal with such an issue, also pulling all these developments inside a probability territory (Rao and Ballard, 2005; Seth, 2014) in which a lot of development still needs to be done. Moreover, the way this framework can be extended to agents exhibiting dynamical effects, e.g., when performing kinematic or dynamical control, must still be investigated. This requires some clarification about the structure and role of time in the sensorimotor experience, a point which is still largely eluded in the SMCT context. Finally, actions can also be noisy, and the question of their repeatability over time needs to be addressed so as to face realistic conditions. This poses significant challenges in the SMCT context of minimal *a priori* knowledge outlined in the present contribution. However, ongoing exploratory work tends to show that topological structure grounding some continuity of the sensorimotor experience can be found as a contingency in said naive context. All these paths constitute future promising works in the field and will undoubtedly extend the scope of these approaches to naive adaptive and robust agents able to build by themselves their own understanding of their interaction with their environment.

## Data Availability Statement

The raw data supporting the conclusions of this article will be made available by the authors, without undue reservation.

## Author Contributions

Most of this work has been conducted by J-MG, supervised mainly by SA, and advised by BG. All authors contributed to the article and approved the submitted version.

## Conflict of Interest

The authors declare that the research was conducted in the absence of any commercial or financial relationships that could be construed as a potential conflict of interest.

## Footnotes

^1^In all the following, the map ψ_C_ will be shortened to ψ when there is no ambiguity, consistently with the initial definition of the sensorimotor map of the agent recalled in section 2.1.1.
