# Supplementary material for: A Formal Account of Structuring Motor Actions With Sensory Prediction for a Naive Agent
Source: Front Robot AI. 2020 Dec 1;7:561660. doi: 10.3389/frobt.2020.561660 (PMC7805968; doi:10.3389/frobt.2020.561660)
Supplement: Supplementary file 1 [file Data_Sheet_1.pdf]

Supplementary Material

APPENDIX - PROOFS FOR THE STATEMENTS OF SECTION 3 AND 4

In the following proofs, we will assume

$$\big(\forall \mathbf{b} \in \mathscr{B}, \forall \epsilon \in \mathscr{E},\ \psi_{\mathscr{C}}(a'\mathbf{b}, \epsilon) = \psi_{\mathscr{C}}(a\mathbf{b}, \epsilon)\big) \Rightarrow a = a' \tag{S1}$$

and

$$\forall c, c' \in \mathscr{C},\ F_c = F_{c'} \Rightarrow c = c'. \tag{S2}$$

These assumptions are of minimal importance for two reasons

- Eq. (S2) only mandates that any two distinct sensels have different receptive fields *if only one time*, while Eq. (S1) asks for actions to have no difference (as denoted by $=$) except that which can be assessed by the sensory capabilities $\psi_{\mathscr{C}}$. These conditions only fail to hold in very particular cases and can be found to be true in the presented examples.
- In any case where they indeed *fail* to hold, the exact same results can be found with suitable equivalence relations for actions (for Eq. (S1)) and sensels (for Eq. (S2)) at the cost of more loaded notations.

Therefore these conditions only serve as a way to streamline the presentation of the results with an at most negligible impact on generality.

1 Equivalency between conservative and permutative

Here is provided a proof that conservative actions can be described as permutations of sensels, as discussed in Section 3.3.1.

PROPOSITION 1. *Let a be a conservative action $\in \mathcal{A}$, there exists a unique map*

$$\begin{aligned} \sigma_a : \mathscr{C} &\to \mathscr{C} \\ c &\mapsto c' \end{aligned} \tag{S3}$$

such that

$$\sigma_a(c) = c' \Leftrightarrow c \xrightarrow{a} c' \tag{S4}$$

PROOF. Let $a \in \mathcal{A}$ conservative and $c \in \mathscr{C}$. By conservativity $\exists c' \in \mathscr{C}$ such that $c \xrightarrow{a} c'$. Let $c'' \in \mathscr{C}$ such that $c \xrightarrow{a} c''$, then

$$\forall \mathbf{b} \in \mathscr{B}, F_c(\mathbf{b}) = F_{c'}(a\mathbf{b}) \text{ and } F_c(\mathbf{b}) = F_{c''}(a\mathbf{b})$$

so that

$$\forall \mathbf{b} \in \mathscr{B}, F_{c'}(a\mathbf{b}) = F_{c''}(a\mathbf{b}).$$

But $a : \mathscr{B} \to \mathscr{B}$ must be surjective because it is bijective, so that *all* $\mathbf{b} \in \mathscr{B}$ can be written a**b'** for some **b'** $\in \mathscr{B}$. Therefore $F_{c'} = F_{c''}$, from which $c' = c''$: successor sensels are necessarily unique. We therefore declare σ_a to be the map that takes each sensel $c \in \mathscr{C}$ to its *unique* successor sensel.

PROPOSITION 2. *For any conservative action $a \in \mathcal{A}$, its successor map σ_a is bijective.*

PROOF. Let a be a conservative action, and let $c, c' \in \mathscr{C}$ be sensels such that $\sigma_a(c) = \sigma_a(c')$. From this it follows that

$$\forall \mathbf{b} \in \mathscr{B}, F_c(\mathbf{b}) = F_{c''}(a\mathbf{b}) \text{ and } F_{c'}(\mathbf{b}) = F_{c''}(a\mathbf{b})$$

for some common successor $c'' \in \mathscr{C}$. But it entails in particular

$$\forall \mathbf{b} \in \mathscr{B}, F_c(\mathbf{b}) = F_{c'}(\mathbf{b})$$

that is $F_c = F_{c'}$, which further yields $c = c'$: σ_a is injective.
From injectivity of σ_a, it follows that $|\sigma_a(\mathscr{C})| = |\mathscr{C}|$. But because $\mathscr{C}$ is finite it in turns follows from this equality that $\sigma_a(\mathscr{C}) = \mathscr{C}$, i.e. σ_a is also surjective.

2 Conversing of conserving

We provide here the proof, as used in Section 3.3.1 and following, that conservative actions are themselves a subgroup of $\mathcal{A}$ for its succession operation.

PROPOSITION 3. *Let $\mathcal{A}_\mathscr{C} \subset \mathcal{A}$ be the subset of all conservative actions. Then $\mathcal{A}_\mathscr{C}$ is in fact a* subgroup *of $\mathcal{A}$.*

PROOF. $\mathcal{A}_\mathscr{C} \subset \mathcal{A}$ by its very definition, therefore we only need prove it is actually a group.

- $\forall c \in \mathscr{C},\ c \overset{e}{\to} c$ with e the identity action: e is conservative.
- Let a and a' be conservative actions, and $c \in \mathscr{C}$: since $a \in \mathcal{A}_\mathscr{C}$, $\exists c' \in \mathscr{C}$ such that $c \overset{a}{\to} c'$. But since $a' \in \mathcal{A}_\mathscr{C}$ too, there also exists $c'' \in \mathscr{C}$ verifying $c' \overset{a'}{\to} c''$, so that finally

  $$\forall c \in \mathscr{C}, \forall a, a' \in \mathcal{A}_\mathscr{C},\ \exists c'' \text{ such that } c \overset{a'a}{\to} c''$$

  that is $a'a$ is conservative itself.
- Let $a \in \mathcal{A}_\mathscr{C}$ and let σ_a be its successor map $\mathscr{C} \to \mathscr{C}$. $\forall c \in \mathscr{C}$ since σ_a is surjective (see proof in 1) we have $c = \sigma_a(c')$ for some $c' \in \mathscr{C}$, or equivalently

  $$\forall c \in \mathscr{C},\ \exists c' \text{ such that } c' \overset{a}{\to} c.$$

  Finally, since $c' \overset{a}{\to} c \Leftrightarrow c \overset{a^{-1}}{\to} c'$ it follows that a^{-1} is conservative too.

3 Conservation or prediction, it is all the same

Here is provided a proof that mapping conservative actions a to their respective *sensory prediction functions* Π_a provides a group isomorphism, as per Equation (15). To this end, let us recall the essential property of these functions:

$$\forall \mathbf{b} \in \mathscr{B}, \forall \epsilon \in \mathscr{E},\ \psi_\mathscr{C}(a\mathbf{b}, \epsilon) = \Pi_a(\psi_\mathscr{C}(\mathbf{b}, \epsilon)). \tag{S5}$$

From this we get:

PROPOSITION 4. *The map*

$$\begin{aligned} \Pi\colon \mathcal{A}_{\mathscr{C}} &\to \mathrm{Bij}(\mathcal{S}) \\ a &\mapsto \Pi_a \end{aligned} \tag{S6}$$

is a group morphism. Moreover it is injective, so that it induces a group isomorphism $\mathcal{A}_{\mathscr{C}} \cong \Pi(\mathcal{A}_{\mathscr{C}})$.

PROOF. Let a, a' be two conservative actions, we have

$$\begin{aligned} &\forall \mathbf{b} \in \mathscr{B}, \forall \epsilon \in \mathscr{E}, \\ &\Pi_{a'^{-1}a}(\psi_{\mathscr{C}}(\mathbf{b}, \epsilon)) = \psi_{\mathscr{C}}(a'^{-1}a\mathbf{b}, \epsilon) \\ &\qquad = \Pi_{a'^{-1}}(\psi(a\mathbf{b}, \epsilon)) \\ &\qquad = \Pi_{a'^{-1}}(\Pi_a(\psi(\mathbf{b}, \epsilon))) \\ &\qquad = (\Pi_{a'^{-1}} \circ \Pi_a)(\psi(\mathbf{b}, \epsilon)) \end{aligned}$$

so that $\Pi(a'^{-1}a) = \Pi(a'^{-1}) \circ \Pi(a)$: Π is a group morphism.

Now let $a, a' \in \mathcal{A}_{\mathscr{C}}$ such that $\Pi_a = \Pi_{a'}$. It follows from Equation (S5) that

$$\forall \mathbf{b} \in \mathscr{B}, \forall \epsilon \in \mathscr{E},\ \psi_{\mathscr{C}}(a'\mathbf{b}, \epsilon) = \psi_{\mathscr{C}}(a\mathbf{b}, \epsilon)$$

so that under hypothesis (S1) Π is indeed injective.

4 Convergence of experiment 1

This part is devoted to the proof of the relevance of Equation (17) in Section 4.2.1, that is the convergence of matrices M_a towards the associated permutation matrices M_{σ_a} for all conservative actions a.

LEMMA 1. *For any coefficient* $m_{a_{i,j}}$ *of* M_{σ_a}*, the associated sequence* $\left(m_{a_{i,j}}[t_n]\right)_n$ *of values taken in* $\left(M_a[t_n]\right)_n$ *during exploration is nonincreasing with values in* $\{0, 1\}$.

Proof. Let us consider an arbitrary timestep $t_n,\ n \in \mathbb{N}$ in the exploration. If a is not drawn at this timestep, then

$$m_{a_{i,j}}[t_{n+1}] = m_{a_{i,j}}[t_n] \leq m_{a_{i,j}}[t_n].$$

If it is instead chosen, assuming $m_{a_{i,j}}[t_n] \in \{0, 1\}$ then as per the update rule of M_a, either

- $m_{a_{i,j}}[t_n] = 0$ and then $m_{a_{i,j}}[t_{n+1}] = 0$ too,
- or $m_{a_{i,j}}[t_n] = 1$ and $m_{a_{i,j}}[t_{n+1}] \in \{0, 1\}$

so that the lemma follows by induction on n.

LEMMA 2. *For any coefficient* $m_{a_{i,j}} = 1$ *in* M_{σ_a}*, the associated sequence* $\left(m_{a_{i,j}}[t_n]\right)_n$ *is constant with value* $m_{a_{i,j}}[t_n] = 1$.

Proof. At any timestep t_n of the exploration, if a is not chosen then $m_{a_{i,j}}[t_{n+1}] = m_{a_{i,j}}[t_n]$.
If it is instead drawn, then by Equation (11) we know that

$$\mathrm{s}_j[t_{n+1}] = \mathrm{s}_i[t_n]$$

because $m_{a_{i,j}} = 1$ implies that $j = \sigma_a(i)$ as per the definition of M_{σ_a}. Then by the update rule of $M_a[t_n]$, $m_{a_{i,j}}[t_{n+1}] = 1$.
The lemma then follows by induction on n.

We now proceed with the last part of our argument, that is showing that coefficients of the empirical matrices M_{a_k} which do not correspond to successor sensel pairs will actually be nulled during exploration. This specific part is provided in the specific case of the simulated experiment presented, allowing us to formulate the relevant equations in the vector geometry of $\mathcal{X} = \mathbb{R}^2$. The same idea could also be adapted for generalized spaces $\mathcal{X}$, $\mathscr{B}$ and actions $\mathcal{A}_{\text{init}}$, more in line with the previous theoretical descriptions. However, such a development is out of the scope of this contribution.

Let us define

$$\forall \theta \in \mathbb{R},\ R_\theta = \begin{pmatrix} \cos(\theta) & -\sin(\theta) \\ \sin(\theta) & \cos(\theta) \end{pmatrix}$$

the matrix corresponding to the rotation in $\mathbb{R}^2$ of angle θ. As per the definitions provided for our particular example, we may assume the properties:

$$\forall c \in \mathscr{C}, \forall (x, y, \vec{\theta}) \in \mathscr{B},\ F_c(x, y, \vec{\theta}) = \begin{pmatrix} x \\ y \end{pmatrix} + R_\theta F_c(0, 0, \vec{0})$$

and

$$\forall a \in \mathcal{A}_{\text{init}},\ \exists \vec{u}_a = \begin{pmatrix} x_a \\ y_a \end{pmatrix} \in \mathbb{R}^2, \exists \theta_a \in \mathbb{R}$$
$$\text{such that } \forall \mathbf{b} = (x, y, \vec{\theta}) \in \mathscr{B},\ a\mathbf{b} = (x', y', \vec{\theta'})$$
$$\text{where } \begin{pmatrix} x' \\ y' \end{pmatrix} = \begin{pmatrix} x \\ y \end{pmatrix} + R_\theta \begin{pmatrix} x_a \\ y_a \end{pmatrix} \text{ and } \theta' = \theta + \theta_a.$$

Therefore we have

LEMMA 3. *Let $a \in \mathcal{A}_{\text{init}}$, $c, c' \in \mathscr{C}$. There exists a unique vector $\vec{d}_{a,c,c'} \in \mathbb{R}^2$ such that*

$$\forall \boldsymbol{b} = (x, y, \vec{\theta}) \in \mathscr{B},\ \overrightarrow{F_c(a\boldsymbol{b})F_{c'}(\boldsymbol{b})} = R_\theta \vec{d}_{a,c,c'}.$$

PROOF. Let $\mathbf{b} = (x, y, \vec{\theta}) \in \mathscr{B}$. We therefore have

1. $F_{c'}(\mathbf{b}) = \begin{pmatrix} x \\ y \end{pmatrix} + R_\theta F_{c'}(0, 0, \vec{0})$,
2. $F_c(a\mathbf{b}) = F_c\left(\begin{pmatrix} x \\ y \end{pmatrix} + R_\theta \begin{pmatrix} x_a \\ y_a \end{pmatrix}, \overrightarrow{\theta + \theta a}\right)$

$$= \begin{pmatrix} x \\ y \end{pmatrix} + R_\theta \begin{pmatrix} x_a \\ y_a \end{pmatrix} + R_{\theta+\theta_a} F_c(0, 0, \vec{0})$$

so that

$$\overrightarrow{F_c(a\mathbf{b})F_{c'}(\mathbf{b})} = R_\theta \left(F_{c'}(0) - \begin{pmatrix} x_a \\ y_a \end{pmatrix} - R_{\theta_a} F_c(0) \right).$$

which proves taking $\vec{d}_{a,c,c'} = \left(F_{c'}(0) - \begin{pmatrix} x_a \\ y_a \end{pmatrix} - R_{\theta_a} F_c(0) \right)$ satisfies the property.

It should be noted that $\vec{d}_{a,c,c'}$ captures some geometry of conservation: indeed, from the definition of $\stackrel{a}{\to}$ it can easily be shown that

$$\forall a \in_{\text{init}},\ \forall c, c' \in \mathscr{C},\ \left(c \stackrel{a}{\to} c' \Leftrightarrow \vec{d}_{a,c,c'} = 0 \right). \tag{S7}$$

This greatly serves the conclusion of our argument with

PROPOSITION 5. *Let $a \in \mathcal{A}_{\text{init}}$, $c_i, c_j \in \mathscr{C}$. Then for given environment configuration $\epsilon \in \mathscr{E}$ the two statements*

1. *There exists an absolute configuration **b** $\in \mathscr{B}$ such that if **b**$[t_n] =$ **b** and $a[t_n] = a$ for some $n \in \mathbb{N}$, then $m_{a_{i,j}}[t_k] = 0\ \forall k \geq n+1$*
2. *ϵ is not doubly periodic with periods $\vec{d}_{a,c_i,c_j}$ and $R_{\frac{\pi}{2}}\vec{d}_{a,c_i,c_j}$*

are equivalent.

PROOF.

- Assume that ϵ is both $\vec{d}_{a,c_i,c_j}$- and $R_{\frac{\pi}{2}}\vec{d}_{a,c_i,c_j}$-periodic. Let $n \in \mathbb{N}$ such that $a[t_n] = a$, let $\mathbf{b} = \mathbf{b}[t_n]$. We have $\mathrm{s}_i[t_n] = \epsilon(F_c(\mathbf{b}[t_n]))$ and $\mathrm{s}_j[t_{n+1}] = \epsilon(F_{c'}(\mathbf{b}[t_{n+1}])) = \epsilon(F_c(\mathbf{b}[t_n]) + R_\theta \vec{d}_{a,c_i,c_j})$. But since $\theta \in \left\{0, \frac{\pi}{2}, \pi, \frac{3\pi}{2}\right\}$, $R_\theta = \pm I_2$ or $R_\theta = \pm R_{\frac{\pi}{2}}$. Therefore by periodicity of ϵ we have $\mathrm{s}_j[t_{n+1}] = \mathrm{s}_i[t_n]$, from which by induction on n we get $\forall n \in \mathbb{N},\ m_{a_{i,j}}[t_n] = 1$: 1) ⇒ 2).
- Without loss of generality, let us assume that ϵ is not $\vec{d}_{a,c_i,c_j}$-periodic (if it is instead only not $R_{\frac{\pi}{2}}\vec{d}_{a,c,c'}$-periodic, the same argument follows up to a rotation).
Let $X_0 \in \mathbb{R}^2$ such that $\epsilon(X_0) \neq \epsilon(X_0 + \vec{d}_{a,c_i,c_j})$, $\mathbf{b}_0 = (x, y, \vec{0}) \in \mathscr{B}$ such that $F_c(\mathbf{b}_0) = X_0$. By definition $F_{c'}(a\mathbf{b}_0) = X_0 + \vec{d}_{a,c_i,c_j}$ so that if $\mathbf{b}[t_n] = \mathbf{b}_0$ for some $t_n \in \mathbb{N}$, $\mathrm{s}_j[t_{n+1}] \neq \mathrm{s}_i[t_n]$. From the update rule of M_a we then get $m_{a_{i,j}}[t_{n+1}] = 0$, which by Lemma 1 concludes the proof.

Finally, simultaneously applying this proof to *all* actions and pair of sensels of the agent has us deduce:

COROLLARY. *If $\epsilon : \mathbb{R}^2 \to \mathcal{P}$ is aperiodic, then there exists a sequence of drawings of actions $(a[t_n])_{n\in\mathbb{N}}$ such that*

$$\forall a_k \in \mathcal{A}_{\text{init}},\ \lim_n M_{a_k}[t_n] = M_{\sigma_{a_k}}.$$

While the converse strictly speaking is not true, we can see from the preliminary lemma that problems in the algorithm arise from very particular periodicity properties which relate to the geometry of (receptive fields of) sensels. It therefore should be noted already how most experiments in live specimens made use of specifically engineered symmetric and *periodic* environments to try and impair the development of perception (Held and Hein, 1963). Future works could expand on the effects of such "pathological" environment configurations on the proposed algorithm.

REFERENCES

Held, R. and Hein, A. (1963). Movement-produced stimulation in the development of visually guided behavior. *Journal of comparative and physiological psychology* 56, 872–6. doi:10.1037/h0040546
